# Supplementary material for: Role and knowledge of nurses in the management of non-communicable diseases in Africa: A scoping review
Source: PLoS One. 2024 Apr 18;19(4):e0297165. doi: 10.1371/journal.pone.0297165 (PMC11025970; doi:10.1371/journal.pone.0297165)
Supplement: S1 Table — (DOCX) [file pone.0297165.s001.docx]

| **Supplementary Table 1.** **Search strategy for each database included in the review** | | |  |
| --- | --- | --- | --- |
| Database | Equation | Articles identified till 02-2022 | Articles identified during update (02-2022 to 04-2023) |
| Pubmed | ((nurs*[MeSH Terms]) OR (nurs*[Title/Abstract])) AND ((noncommunicable disease[MeSH Terms]) OR (chronic disease[MeSH Terms]) OR (Non-transmissible disease[MeSH Terms]) OR (diabetes[MeSH Terms]) OR (cancer[MeSH Terms]) OR (asthma[MeSH Terms]) OR (chronic obstructive pulmonary disease[MeSH Terms]) OR (chronic respiratory failure[MeSH Terms]) OR (chronic kidney failure[MeSH Terms]) OR (obesity[MeSH Terms]) OR (undernutrition[MeSH Terms]) OR (chronic hepatitis[MeSH Terms]) OR (nonalcoholic fatty liver disease[MeSH Terms]) OR (non-alcoholic steatohepatitis[MeSH Terms]) OR (chronic pain[MeSH Terms]) OR (epilepsy[MeSH Terms]) OR (stroke[MeSH Terms]) OR (high blood pressure[MeSH Terms]) OR (chronic heart failure[MeSH Terms]) OR (coronary syndrome[MeSH Terms]) OR (Alzheimer disease[MeSH Terms]) OR (Parkinson disease[MeSH Terms]) OR (dementia[MeSH Terms]) OR (schizophrenia[MeSH Terms]) OR (depression[MeSH Terms]) OR (psychosis[MeSH Terms]) OR (bipolar disorder[MeSH Terms]) OR (Noncommunicable disease[Title/Abstract]) OR (chronic disease[Title/Abstract]) OR (Non-transmissible disease[Title/Abstract) OR (diabetes[Title/Abstract]) OR (cancer[Title/Abstract]) OR (asthma[Title/Abstract]) OR (chronic obstructive pulmonary disease[Title/Abstract]) OR (chronic respiratory failure[Title/Abstract]) OR (chronic kidney failure[Title/Abstract]) OR (obesity[Title/Abstract]) OR (undernutrition[Title/Abstract]) OR (chronic hepatitis[Title/Abstract]) OR (nonalcoholic fatty liver disease[Title/Abstract]) OR (non-alcoholic steatohepatitis[Title/Abstract]) OR (chronic pain[Title/Abstract]) OR (epilepsy[Title/Abstract]) OR (stroke[Title/Abstract]) OR (high blood pressure[Title/Abstract]) OR (chronic heart failure[Title/Abstract]) OR (coronary syndrome[Title/Abstract]) OR (Alzheimer disease[Title/Abstract]) OR (Parkinson disease[Title/Abstract]) OR (dementia[Title/Abstract]) OR (schizophrenia[Title/Abstract]) OR (depression[Title/Abstract]) OR (psychosis[Title/Abstract]) OR (bipolar disorder[Title/Abstract])) AND (((Africa[MeSH Terms]) OR (Africa[Title/Abstract])) ((Africa[MeSH Terms]) OR (Algeria[MeSH Terms]) OR (Angola[MeSH Terms]) OR (Bangladesh[MeSH Terms]) OR (Benin[MeSH Terms]) OR (Botswana[MeSH Terms]) OR (Burkina Faso[MeSH Terms]) OR (Burundi[MeSH Terms]) OR (Cabo Verde[MeSH Terms]) OR (Cameroon[MeSH Terms]) OR (Central African Republic[MeSH Terms]) OR (Chad[MeSH Terms]) OR OR (Comoros[MeSH Terms]) OR (Congo Dem. Rep[MeSH Terms]) OR (Congo, Rep[MeSH Terms]) OR (Cote d'Ivoire[MeSH Terms]) OR (Djibouti[MeSH Terms]) OR (Egypt, Arab Rep[MeSH Terms]) OR (Equatorial Guinea[MeSH Terms]) OR (Eritrea[MeSH Terms]) OR (Eswatini[MeSH Terms]) OR (Ethiopia[MeSH Terms]) OR (Gabon[MeSH Terms]) OR (Gambia[MeSH Terms]) OR (Ghana[MeSH Terms]) OR (Guinea[MeSH Terms]) OR (Guinea-Bissau[MeSH Terms]) OR (Jamaica[MeSH Terms]) OR (Kenya[MeSH Terms]) OR (Lesotho[MeSH Terms]) OR (Liberia[MeSH Terms]) OR (Madagascar[MeSH Terms]) OR (Malawi[MeSH Terms]) OR (Mali[MeSH Terms]) OR (Mauritania[MeSH Terms]) OR (Mauritius[MeSH Terms]) OR (Morocco[MeSH Terms]) OR (Mozambique[MeSH Terms]) OR (Myanma[MeSH Terms]) OR (Namibia[MeSH Terms]) OR (Niger[MeSH Terms]) OR (Nigeri[MeSH Terms]) OR (Rwanda[MeSH Terms]) OR (Sao Tome and Principe[MeSH Terms]) OR (Senegal[MeSH Terms]) OR (Sierra Leone[MeSH Terms]) OR (Somalia[MeSH Terms]) OR (South Africa[MeSH Terms]) OR (South Sudan[MeSH Terms]) OR (Sudan[MeSH Terms]) OR (Suriname[MeSH Terms]) OR (Tanzania[MeSH Terms]) OR (Togo[MeSH Terms]) OR (Tunisia[MeSH Terms]) OR (Turkey[MeSH Terms]) OR (Uganda[MeSH Terms]) OR (Zambia[MeSH Terms]) OR (Zimbabwe[MeSH Terms]))). | 1127 | 99 |
| PsycINFO | (MAINSUBJECT.EXACT("Nurse") OR MAINSUBJECT.EXACT("Nurse led") OR MAINSUBJECT.EXACT("nursing") OR “nurse” OR “nurse led”) AND ((MAINSUBJECT.EXACT("chronic disease") OR MAINSUBJECT.EXACT("noncommunicable disease") OR “chronic disease” OR “noncommunicable disease” OR “cancer” OR “asthma” OR “chronic obstructive pulmonary disease” OR “chronic respiratory failure” OR “chronic kidney failure” OR “obesity” OR “undernutrition” OR “chronic hepatitis” OR “nonalcoholic fatty liver disease” OR “non-alcoholic steatohepatitis” OR “chronic pain” OR “epilepsy” OR “stroke” OR “high blood pressure” OR “chronic heart failure” OR “coronary syndrome” OR ”Alzheimer disease” OR “Parkinson disease” OR “dementia” OR “schizophrenia” OR “depression” OR “psychosis” OR “bipolar disorder”) AND "Africa" | 15 | 1 |
| CINAHL | ((MH "nurse") OR (MH "nurse led") OR (MH “nursing) OR “nursing” OR “nurse led” OR “nurse”) AND ((MH “noncommunicable disease”) OR (MH “Chronic disease”) OR “chronic disease” OR “noncommunicable disease” OR “cancer” OR “asthma” OR “chronic obstructive pulmonary disease” OR “chronic respiratory failure” OR “chronic kidney failure” OR “obesity” OR “undernutrition” OR “chronic hepatitis” OR “nonalcoholic fatty liver disease” OR “non-alcoholic steatohepatitis” OR “chronic pain” OR “epilepsy” OR “stroke” OR “high blood pressure” OR “chronic heart failure” OR “coronary syndrome” OR ”Alzheimer disease” OR “Parkinson disease” OR “dementia” OR “schizophrenia” OR “depression” OR “psychosis” OR “bipolar disorder”) AND ((MH “Africa”) OR “Africa”) | 1210 | 4 |
| Web of Science | ((ALL=(nurs*)) AND ALL=(africa)) AND (ALL=(noncommunicable disease) OR ALL=(chronic disease)) | 527 | 119 |
| Embase | ('nurse' OR 'nurse-led intervention') AND ('noncommunicable disease' OR 'chronic disease' OR 'non-transmissible disease' OR 'ncd' OR 'diabetes' OR 'cancer' OR 'asthma' OR 'chronic obstructive pulmonary disease' OR 'chronic respiratory failure' OR 'chronic kidney failure' OR 'obesity' OR 'undernutrition' OR 'chronic hepatitis' OR 'nonalcoholic fatty liver disease' OR 'non-alcoholic steatohepatitis' OR 'chronic pain' OR 'epilepsy' OR 'stroke' OR 'hight blood pressure' OR 'chronic arteriopathy' OR 'chronic heart failure' OR 'coronary syndrom' OR 'alzheimer' OR 'parkinson' OR 'dementia' OR 'schizophrenia' OR 'depression' OR 'psychosis' OR 'bipolar disorder') AND ('africa') | 438 | 117 |
