# Supplementary material for: Role and knowledge of nurses in the management of non-communicable diseases in Africa: A scoping review
Source: PLoS One. 2024 Apr 18;19(4):e0297165. doi: 10.1371/journal.pone.0297165 (PMC11025970; doi:10.1371/journal.pone.0297165)
Supplement: S2 Table — Studies were classified according to disease type and in chronological order. (DOCX) [file pone.0297165.s002.docx]

| **S3 Table. Downs and Black assessment for included studies.** | | | | | |
| --- | --- | --- | --- | --- | --- |
| First Author, Year - Location | Report /10 | External validity /3 | Internal Validity – Bias & Confounding /13 | Power /5 | Total |
| Cardiovascular disease | | | | | |
| Bassett *et al.*, 1990 - Zimbabwe | 6 | 1 | 4 | 0 | 11 |
| Kengne, 2009 - Cameroon | 7 | 2 | 4 | 0 | 13 |
| Kwan, 2013 - Rwanda | 7 | 3 | 4 | 0 | 14 |
| Lulebo, 2017 - Democratic Republic of Congo | 7 | 3 | 4 | 0 | 14 |
| Blackstone, 2017 - Ghana | 6 | 3 | 4 | 0 | 13 |
| Gyamfi, 2017 - Ghana | 7 | 3 | 7 | 0 | 17 |
| Wahab, 2017 - Nigeria | 7 | 3 | 7 | 0 | 17 |
| Shanko, 2018 - Ethiopia | 6 | 3 | 4 | 0 | 13 |
| Sarfo, 2018 - Ghana | 10 | 3 | 10 | 0 | 23 |
| Ogedegbe, 2018 - Ghana | 10 | 2 | 10 | 0 | 22 |
| Bolarinwa, 2019 - Nigeria | 7 | 3 | 7 | 0 | 17 |
| Adler*,* 2019 - South Africa | 7 | 3 | 7 | 0 | 17 |
| Vedanthan, 2020 - Kenya | 7 | 3 | 4 | 0 | 14 |
| Spies, 2021 - Zambia | 7 | 3 | 4 | 0 | 14 |
| Metabolic diseases | | | | | |
| Goodman, 1997 – South Africa | 7 | 3 | 3 | 0 | 13 |
| Koura, 2001 - Egypt | 6 | 3 | 3 | 0 | 12 |
| Louwagie, 2002 - South Africa | 9 | 3 | 6 | 5 | 23 |
| Van de Sande, 2007 - South Africa | 9 | 3 | 6 | 5 | 23 |
| Gill, 2008 – South Africa | 7 | 3 | 6 | 5 | 21 |
| Kengne, 2009 - Cameroon | 10 | 3 | 7 | 5 | 25 |
| Price, 2011 - South Africa | 7 | 2 | 4 | 0 | 13 |
| Matimba, 2016 - Zimbabwe | 7 | 3 | 4 | 0 | 14 |
| Essien, 2017 -Nigeria | 7 | 3 | 7 | 0 | 17 |
| Ndayisaba, 2017 - Rwanda | 6 | 3 | 4 | 0 | 14 |
| Garanet, 2018 - Mali & Burkina faso | 7 | 3 | 4 | 0 | 14 |
| Hailu, 2021 - Ethiopia | 9 | 3 | 5 | 0 | 17 |
| Asante*,* 2020 - Ghana | 10 | 3 | 10 | 0 | 23 |
| Tamiru, 2023 - Ethiopia | 8 | 3 | 8 | 5 | 24 |
| Neurological disease | | | | | |
| Adamolekun, 1999 – Zimbabwe | 8 | 3 | 3 | 0 | 15 |
| Adamolekun, 2000 - Zimbabwe | 6 | 3 | 4 | 5 | 18 |
| Kengne, 2008 - Cameroon | 10 | 3 | 7 | 5 | 25 |
| Rhodat, 2015 - Namibia | 7 | 3 | 4 | 0 | 14 |
| Pierpoint, 2020 - South Africa | 7 | 3 | 4 | 0 | 14 |
| Knight, 2020 - South Africa | 9 | 2 | 5 | 0 | 16 |
| Cancers | | | | | |
| Odusanya, 2001 - Nigeria | 6 | 3 | 3 | 0 | 12 |
| Ayinde, 2003 - Nigeria | 6 | 3 | 4 | 0 | 13 |
| Anya, 2005 - Nigeria | 6 | 3 | 4 | 0 | 13 |
| Sangwa-Lugoma, 2006 - Democratic Republic of Congo | 9 | 3 | 6 | 0 | 18 |
| Udigwe, 2006 - Nigeria | 6 | 3 | 3 | 0 | 12 |
| Ibrahim, 2009 - Nigeria | 7 | 2 | 4 | 0 | 13 |
| Akhigbe, 2009 - Nigeria | 7 | 3 | 3 | 0 | 13 |
| Awodele, 2009 - Nigeria | 7 | 3 | 3 | 0 | 13 |
| Awodele, 2011 - Nigeria | 7 | 2 | 4 | 0 | 13 |
| Ghanem, 2011 - Morocco | 7 | 2 | 4 | 0 | 13 |
| Urasa, 2011 - Tanzania | 7 | 3 | 4 | 0 | 14 |
| Bello, 2011 - Nigeria | 7 | 2 | 4 | 0 | 13 |
| Arulogun, 2012 - Nigeria | 7 | 3 | 4 | 0 | 14 |
| Rwamugira, 2012 - South Africa | 8 | 3 | 4 | 0 | 15 |
| Moon, 2012 - Mozambique | 7 | 3 | 4 | 0 | 14 |
| Wamai, 2013 - Cameroon | 7 | 3 | 4 | 0 | 14 |
| Mwanahamuntu*.*, 2013 - Zambia | 7 | 3 | 4 | 0 | 14 |
| Manga, 2015 - Cameroon | 7 | 3 | 4 | 0 | 14 |
| Firnhaber, 2015 - South Africa | 7 | 3 | 4 | 0 | 14 |
| DeGregorio, 2017 - Cameroon | 7 | 3 | 4 | 0 | 14 |
| Asgary, 2016 - Ghana | 7 | 3 | 4 | 0 | 14 |
| Dickerson, 2017 - South Africa | 6 | 3 | 4 | 0 | 13 |
| Pace, 2018 - Rwanda | 7 | 3 | 7 | 0 | 17 |
| Dareng, 2018 - Nigeria | 7 | 3 | 4 | 0 | 14 |
| Ndikom, 2019 - Nigeria | 7 | 3 | 7 | 0 | 17 |
| Asgary, 2019 - Ghana | 7 | 3 | 7 | 0 | 17 |
| Pruitt, 2020 - Nigeria | 7 | 3 | 7 | 0 | 17 |
| O’Neil, 2021 - Eswatini | 9 | 3 | 5 | 0 | 17 |
| Mkhonta, 2021 - Swaziland | 7 | 3 | 4 | 0 | 14 |
| Domgue, 2020 - Cameroon | 7 | 3 | 4 | 0 | 14 |
| Obol, 2021 - Uganda | 9 | 3 | 5 | 0 | 17 |
| Mango, 2022 –  Nigeria | 10 | 3 | 9 | 0 | 22 |
| Taj, 2022 – Kenya | 8 | 3 | 7 | 0 | 18 |
| Getachew, 2022 - Ethiopia | 9 | 3 | 11 | 5 | 28 |
| Chitha, 2023 – South Africa | 8 | 3 | 8 | 5 | 23 |
| Respiratory diseases | | | | | |
| Kengne, 2008 - Cameroon | 10 | 3 | 6 | 5 | 24 |
| Psychiatric diseases | | | | | |
| Ndetei, 2011 - Kenya | 7 | 3 | 4 | 0 | 14 |
| Adams, 2012 - Tanzania | 7 | 3 | 4 | 0 | 14 |
| Chetty, 2013 - South Africa | 10 | 3 | 9 | 5 | 27 |
| Alonso, 2014 - Sierra Leone | 7 | 3 | 4 | 0 | 14 |
| Wagner, 2016 - Uganda | 7 | 3 | 8 | 5 | 23 |
| Joubert, 2018 - South Africa | 7 | 3 | 4 | 0 | 14 |
| Muga, 2019 - Kenya | 7 | 3 | 4 | 0 | 14 |
| Petersen, 2019 - South Africa | 10 | 3 | 10 | 0 | 23 |
| Kemp, 2020 - South Africa | 9 | 3 | 5 | 0 | 17 |
| Smith, 2020 - Rwanda | 7 | 3 | 4 | 0 | 14 |
| Kathree, 2023 – South Africa | 10 | 3 | 8 | 0 | 21 |
| Chronic kidney diseases | | | | | |
| Gapira, 2020 - Rwanda | 7 | 3 | 5 | 0 | 15 |
| Chronic diseases globally | | | | | |
| Coleman, 1998 - South Africa | 7 | 2 | 3 | 0 | 12 |
| Kengne, 2009 - Cameroon | 5 | 3 | 2 | 0 | 10 |
| Katz, 2009 - South Africa | 7 | 3 | 3 | 0 | 13 |
| Parker, 2011 - South Africa | 7 | 3 | 4 | 0 | 14 |
| Khabala, 2015 - Kenya | 7 | 3 | 4 | 0 | 14 |
| Malan, 2016 - South Africa | 7 | 3 | 4 | 0 | 14 |
| Some, 2016 - Kenya | 7 | 2 | 3 | 0 | 12 |
| Mahomed, 2017 - South Africa | 7 | 3 | 4 | 0 | 14 |
| Sharp, 2020 - Eswatini | 9 | 2 | 5 | 0 | 16 |
| Niyonsenga, 2021 - Rwanda | 7 | 3 | 4 | 0 | 14 |
