# Supplementary material for: Role and knowledge of nurses in the management of non-communicable diseases in Africa: A scoping review
Source: PLoS One. 2024 Apr 18;19(4):e0297165. doi: 10.1371/journal.pone.0297165 (PMC11025970; doi:10.1371/journal.pone.0297165)
Supplement: S3 Table — (DOCX) [file pone.0297165.s003.docx]

| Supplementary table 2 : Description of the included studies | | | | | | | |
| --- | --- | --- | --- | --- | --- | --- | --- |
| **First Author, Year - Location** | **Aim** | **Study Design** | **Disease** | **Sample** | **Results – Role or Knowledge of nurses** | **δ : Facilitators**  **ε : Barriers** | **α : Limitations**  **β : Perspectives** |
| ***Cardiovascular diseases*** | | | | | | | |
| Bassett *et al.*, 1990 - Zimbabwe | To assess the management of patients with HBP in municipal clinics in Harare | Retrospective records analysis | HBP | 437 patients | Followed blood pressure and adapted anti-hypertensive treatment referred only 11 non-controlled HBP. | δ : A nurse was dedicated to HBP care  ε : Noncompliance | α : retrospective analysis  β : nurses could play a central role in HBP management |
| Kengne, 2009 - Cameroon | To implement a nurse-led protocol for the care of hypertension | Prospective non-randomized interventional program | HBP | 2 nurses  454 patients | A clinical management algorithm was designed from international guidelines to assist nurses’ HBP evaluation and drug prescription.  Statistically significant systolic (p<0.001) and diastolic (p<0.001) blood pressure decrease. | δ : nurses training, use of an algorithm, nurse prescription, medical supervision, the program include referral  ε : lack of material | α : unblinded assessment of endpoints, the absence of a comparative arm, inability to assess the levels of other cardiovascular risk factors  β : long follow-up studies are needed |
| Kwan, 2013 - Rwanda | To describe a decentralized strategy for heart failure diagnosis and management and report the clinical epidemiology at district hospitals in rural Rwanda | Retrospective records analysis | Heart failure | 192 patients | Nurses, supervised by physicians, were trained to use simplified diagnostic and  treatment algorithms including echocardiography with diagnoses confirmed by a cardiologist. | δ : mentoring monthly by medical doctors, specialized nurse training theoretical (2 months) and practical (16 days)  ε : lack of material | α : high clinic volume leads to incomplete  clinical and research data recording  β : training nurses, supervised by physicians, in simplified protocols and basic echocardio-  graphy is a promising approach to decentralized chronic disease care |
| **First Author, Year - Location** | **Aim** | **Study Design** | **Disease** | **Sample** | **Results – Role or Knowledge of nurses** | **δ : Facilitators**  **ε : Barriers** | **α : Limitations**  **β : Perspectives** |
| Lulebo, 2017 - Democratic Republic of Congo | To assess the management of hypertension in primary health care settings by using guidelines of the International Forum for Prevention and Control of HBP in Africa | Cross-sectional | HBP | 102 nurses | Less than a quarter of the nurses knew the cut-off values of hypertension, diabetes  and obesity. Merely 14.7 % knew the therapeutic goals for uncomplicated hypertension. Several of the indicators for  immediate referral recommended by International Forum for Prevention and Control of HBP in Africa were unmentioned. The content of patient education was lacking, avoiding stress being the best advice provided to hypertensive patients. The antihypertensive most used were unlikely to be recommended. There is a considerable gap in knowledge and practices among HBP nurses’ management  according to guidelines. | δ : no identified  ε : lack of in job training, non-optimal quality of guidelines | α : small sample, self-reported data collection  β : training on HBP is needed |
| Vedanthan, 2016 - Kenya | To evaluate the feasibility and impact of nurse management of hypertension in Kenya | Qualitative | HBP | 9 nurses  48 stakeholders | Patients identified barriers: asymptomatic nature of hypertension, poor knowledge, and perception of HBP, financial concerns. For nurses, barriers identified: inadequate training of health care personnel, work overload. Regarding health system barriers were access to drugs, high cost of NCD, access to health care resources, limited human resources for health, negative patient - provider relationship, politics, and corruptions.  Facilitators: Patient were satisfied, nurse should be primary providers. Need for nurse’s empowerment and to create integrated care. | δ : good perception of nurses  ε : lack of material, poor knowledge among HBP, lack of material, shortage in nurses | α : limited generalizability, individual data not recorded  β : central involvement of nursers should be a key point for HBP management |
| **First Author, Year - Location** | **Aim** | **Study Design** | **Disease** | **Sample** | **Results – Role or Knowledge of nurses** | **δ : Facilitators**  **ε : Barriers** | **α : Limitations**  **β : Perspectives** |
| Blackstone, 2017 - Ghana | To explore factors that would influence the sustainability of the task-shifting strategy for blood pressure control in Ghana | Mixed method | HPB | 28 nurses | Factors influencing sustainability were limited drug supply, financial support, provision of primary health care, personnel training, and patient-provider communication. The limited supply of antihypertensive medication was considered by nurses as the most important item to address, while providing training for intervention personnel was considered most feasible to address. | δ : training  ε : costs | α : limited generalizability, small sample  β : It is important to examine nurses’ perceptions of factors likely to influence the sustainability of evidence-based, task-shifting interventions |
| Gyamfi, 2017 - Ghana | To assess the effects of the task-shifting strategy for hypertension on nurses’ knowledge and practice of hypertension management and control in Ghana | Mixed method | HBP | 64 nurses | Marked improvement in nurses’ knowledge and practice related to hypertension detection and treatment. At pre-assessment 26.9% of the nurses scored 80% or more on the hypertension knowledge test, this improved significantly to 95.7% post-training. Improvement of interpersonal skills and patient education were also mentioned by the nurses as positive outcomes of participation in the intervention. | δ : training  ε : no identified | α : small sample, study based on self-report  β  : nurses could play an important role in HBP management |
| **First Author, Year - Location** | **Aim** | **Study Design** | **Disease** | **Sample** | **Results – Role or Knowledge of nurses** | **δ : Facilitators**  **ε : Barriers** | **α : Limitations**  **β : Perspectives** |
| Wahab, 2017 - Nigeria | To assess the feasibility and preliminary short-term effects of implementing a nurse-led group clinic centered intervention, i.e., the Nurse Ushered Discussion Group Encounters after Stroke protocol, in a SSA country to address blood pressure management after a recent stroke | Pilot randomized trial | HPB | 35 patients | After training, nurses-led groups were proposed in the intervention group including health education with family, education and skill-building related to stroke warning signs, stroke risk factors, medication management, community resources vs standard care for control group. At the post-intervention clinic, patient retention rate was 100%. In the intervention group, both the systolic and diastolic BPs measured at home were lower than the clinic BPs post-intervention (127 ± 12.88/78.13 ± 19.26 mmHg versus 137.50 ± 23.05/84.06 ± 9.67 mmHg; p = 0.05). However, there was no significant change in clinic blood pressure recordings in both the intervention and control groups. | δ : training  ε : no identified | α : small sample, lack of home BP measurements in the control group which made it impossible to compare home and clinic BP measurement in the intervention and control groups  β  : It is possible to initiate a nurse-led intervention to address BP management among stroke survivors |
| Iwelunmor, 2017 - Ghana) | To explore stakeholders' perception of an on-going evidence-based  task-shifting strategy for hypertension in 32 community health centers and district hospitals in Ghana | Qualitative | HBP | 42 patients  27 nurses  12  site directors | Almost all the nurses viewed the program as potentially helping their clients to improve their blood pressure. They participated in the program because they enjoyed seeing the positive effects of the program and improvements in patient health. They noted that the program was effective in improving lifestyle and healthful behaviors in ways that previous care was not. One nurse described home visits as a critical component particularly for patient’s adherence to medication. The nurses’ involvement in the program contrasted to their typical role at their clinics with hypertensive patients, in  which previously hypertension diagnosis and management was the responsibility of physicians. Nurses were not allowed to treat hypertension. Leadership support was described as an important factor. | δ : role evolution with prescription and diagnosis, availability of material, supportive director  ε : no area dedicated to program | α : small sample, potential desirability bias  β  : need for on-going evaluations of task-shifting strategies to explore key stakeholders’ perceptions |
| **First Author, Year - Location** | **Aim** | **Study Design** | **Disease** | **Sample** | **Results – Role or Knowledge of nurses** | **δ : Facilitators**  **ε : Barriers** | **α : Limitations**  **β : Perspectives** |
| Shanko, 2018 - Ethiopia | To evaluate the changes in systolic and diastolic blood pressures following treatment over a 30 month period in a rural area of southern Ethiopia where nurses and health officers had been previously trained to diagnose, treat and manage non-communicable diseases including HBP | Retrospective records analysis | HBP | 249 patients | After training and equipment, nurses diagnosed and treated HBP following algorithm.  More than half (53.8%) of patients were controlled on monotherapy, the remainder required combination therapy. Significant declines in systolic and diastolic blood pressure were achieved in each blood pressure group except for the lowest pressure groups. | δ : supervision, training, use of algorithm  ε : no identified | α : small sample, no comparative arm, accuracy of BP measurement  β : support the feasibility of using nurses to effectively manage HBP in  rural areas |
| Sarfo, 2018 - Ghana | To test the feasibility and preliminary efficacy of an m-Health technology-enabled, nurse guided intervention in improving blood pressure (BP) control among Ghanaian stroke patients within one month of symptom onset | Pilot randomized controlled study | BP | 60 patients | Nurses guided a specific program in the intervention group (BP measure with smartphone, tailored motivational, intake medication monitoring) vs standard messages with healthy habits.  Systolic BP <140mmHg at month 3 was  found in 20/30 (66.7%) subjects in the intervention arm vs. 14/30 (46.7%) in the control arm (p=0.12). Medication possession ratio scores at month 3 were better in the intervention (0.88 ± 0.40) vs. control (0.64 ± 0.45) arm (p=0.03). | δ : tailored education, use of smartphone  ε : no identified | α : interim analysis, not powered  β : demonstrated the feasibility of implementing an m-health intervention under nurse  guidance aimed at improving BP control after stroke |
| Spies, 2018 - Uganda | To describe nurses’ knowledge, skills, and confidence related to hypertension and to assess the impact of a hypertension education initiative based in transformational leadership | Pre post intervention | HBP | 16 nurses | Nurses had increased knowledge after completion of workshop. Follow up e-mail query revealed the participants had acted on content of workshop and implemented programs in their communities to screen for and provide education related to HBP. Biometric screening of participants found significant risk factors for HBP but less than expected prevalence of HBP | δ : training  ε : no identified | α : no details on biometrics  β : Educating nurses with the ability to lead and provide care in a complex health system is challenging, transformational leadership could enhance their evolutions |
| **First Author, Year - Location** | **Aim** | **Study Design** | **Disease** | **Sample** | **Results – Role or Knowledge of nurses** | **δ : Facilitators**  **ε : Barriers** | **α : Limitations**  **β : Perspectives** |
| Ogedegbe, 2018 - Ghana | To evaluate the comparative effectiveness of provision of health insurance coverage alone versus a nurse-led task shifting strategy for hypertension control plus health insurance coverage on systolic blood pressure reduction among patients with uncontrolled hypertension | Cluster randomized | HBP | 757 patients | After training nurses treat HBP according to WHO CVD package.  85% of patients had 12-month data available.  In intention-to-treat analyses adjusted for clustering, the nurse-led task shifting strategy for hypertension control plus health insurance coverage group had a greater systolic blood pressure reduction (−20.4 mm Hg; 95% CI −25.2 to −15.6) than the health insurance coverage alone group  (−16.8 mm Hg; 95% CI −19.2 to −15.6), with a statistically significant between-group difference of −3.6 mm Hg (95% CI −6.1 to −0.5; p = 0.021). Blood pressure control improved significantly in both groups with a non-significant between group difference of 5.2% (95% CI −1.8% to 12.4%; p = 0.29). | δ : training, use of WHO CVD package (easy-to-follow algorithms, lifestyle counseling, and drug treatment protocols)  ε : lack of policy to prescribe antihypertension drug | α : lack of cost-effectiveness analysis, lack of policy within Ghana Health Service to grant nurses prescribing power for antihypertensive medications, no control arm  β : medico-economics studies on impact of task shifted nurses’ intervention are needed |
| Bolarinwa, 2019 - Nigeria | To develop, implement, and assess the impact of home‑based follow‑up  care on HRQoL of hypertensive patients attending outpatients’ clinics in Ilorin, Nigeria | Randomized controlled trial | HBP | 299 patients | Home-based based follow up care guidelines for nurses were implemented versus standard care.  The between‑group treatment effect was  not statistically significant (p > 0.05), whereas the within‑group treatment effects  were statistically significant for both the intervention and control arms (p < 0.05)  at 6 months. After controlling for age and baseline HRQoL, the intervention  group had an improved physical component of HRQoL than the control group.  The intervention group also had statistically significant improvement in blood pressure control, medication adherence, and symptom counts (p < 0.05). | δ : home based care  ε : no identified | α : modest assessment of the treatment effect  β : measure impact of Home-based based follow up care on BP itself |
| **First Author, Year - Location** | **Aim** | **Study Design** | **Disease** | **Sample** | **Results – Role or Knowledge of nurses** | **δ : Facilitators**  **ε : Barriers** | **α : Limitations**  **β : Perspectives** |
| Adler*,* 2019 - South Africa | To evaluate the effectiveness of the Community-based Hypertension Improvement Project in increasing hypertension control | Cohort | HBP | 8,339 patients | After training nurses managed HBP patient following a BP management package.  After 1 year of intervention, 72% (95% CI:  67% to 77%) of participants had their hypertension under control. Systolic BP was reduced by 12.2 mmHg (95% CI: 14.4 to 10.1) and diastolic BP by 7.5 mmHg (95% CI: 9.9 to 6.1). | δ : insurance coverage, algorithm use, training  ε : no standard procedures and  backup systems to maximize the possibility that patients stay in the program | α : loss of follow up, no control  β: nurses could manage effectively HBP |
| Haykin, 2020 - Ghana | To explore perceptions of nurses regarding their capacity to manage cardiovascular diseases and barriers to implementing the WHO HEARTS package at Community-based Health Planning and Services facilities in the Upper East Region of Ghana | Qualitative | Cardiovascular diseases | 21 nurses  10 nurses’ supervisors | Nurses identified 3 themes: community demand for cardiovascular diseases care, community access to cardiovascular diseases care, and provider capacities to render cardiovascular diseases care.  Nurses and supervisors noted that community members were often unaware of cardiovascular diseases, despite high reported prevalence of risk factors. Community members were unable to travel for care or afford treatment once diagnosed. Nurses lacked relevant training and medications for treating conditions  such as HBP. Respondents recognized the importance of cardiovascular diseases care, expressed interest in acquiring further  training, and emphasized the need to improve ancillary support for primary care operations. | δ : cardiovascular training, provision of essential equipment and pharmaceuticals, community education campaigns and referral and outreach transportation equipment  ε : poor knowledge, poor access to care, gaps in training, logistics, and other structural factors | α : results were subjective linked to qualitative analysis, use of English (not maternal language)  β : WHO HEARTS package implementation could improve cardiovascular diseases management |
| **First Author, Year - Location** | **Aim** | **Study Design** | **Disease** | **Sample** | **Results – Role or Knowledge of nurses** | **δ : Facilitators**  **ε : Barriers** | **α : Limitations**  **β : Perspectives** |
| Gyamfi, 2020 - Ghana | To use the Consolidated Framework for Implementation Research model to assess nurses’ perceptions of the task shifting strategy for blood pressure control in Ghana, and facilitators and challenges to  implementation | Qualitative | HBP | 27 nurses | Three themes emerged: Patient health goal setting-relative priority and positive feedback from nurses, which motivated patients to make healthy behavior changes as a result of their health being a priority; Leadership engagement (i.e., medical directors) which influenced the extent to which nurses were able to successfully implement task shifting strategy in their various facilities, with most directors being very supportive; and availability of resources making it possible to implement the task shifting protocol. | δ : training, support of direction  ε : limited space and personnel time to carry out task shifting strategy duties, lack of material, lack of transports | α : small sample size  β : feedback from staff were essentials to good implementation’s strategy |
| Vedanthan, 2020 - Kenya | To evaluate the effect of a nurse based HBP management program in Kenya | Retrospective data analysis | HBP | 1051 patients | BP decreased significantly from baseline to three months (nurse-managed patients: slope –4.95mmHg/month; clinical officer-managed patients: slope –5.28), with no significant difference between providers groups. Diastolic BP also significantly decreased from baseline to three months with no difference between provider groups. Retention in care at 12 months was 42%. Nurse-managed HBP care can significantly improve BP. | δ : training, mentorship  ε : retention rate | α : lack of a controlled group  β : prospective trial  settings (nurse led HBP management) with improvements in retention in care, this could be an effective strategy for HBP care |
| **First Author, Year - Location** | **Aim** | **Study Design** | **Disease** | **Sample** | **Results – Role or Knowledge of nurses** | **δ : Facilitators**  **ε : Barriers** | **α : Limitations**  **β : Perspectives** |
| Spies, 2021 - Zambia | To identify nurses and other health care providers perceived barriers and facilitators to the provision of hypertension care in Zambia in the outpatient settings. | Mixed method | HBP | 13 nurses | A statistically significant result was found in the health care providers knowledge and attitude toward HBP. Two overarching themes emerged: infrastructure and belief and behaviors.  Infrastructure was defined as barriers and facilitators to hypertension screening and treatment originating from health system sources. The infrastructure subthemes were primarily economically driven and reflective of policy priorities, essential equipment, and medication availability. The belief and behavior’s theme were influenced by the shared culture, environment, and history. Diverse subthemes identified barriers to HBP treatment that were related to culture and context. It also included: stigma, medication myths and misconceptions, herbal medication, diet, and care-seeking behavior. | δ : training  ε : lack of medication, lack of infrastructure, cultural beliefs | α : small sample, monocentric  β : Equipment and medication must be a priority in the allocation of support additional to nurse education. |
| Kgatla, 2021 - South Africa | To explore and describe the experiences of professional nurses in managing cardiovascular diseases in South African rural and peri-urban clinics. | Qualitative | Cardiovascular diseases | 27 nurses | Two major themes emerged from the data: perceived institutional challenges affecting  the management of cardiovascular diseases with sub themes: the shortage of human resources, the shortage of material resources, the expectations that nurses perform duties outside their scope of practice. The second theme was nurses’ perceptions of patient challenges that impede the effective  management of cardiovascular diseases with sub themes: patients experience long hours waiting at facilities, lack of self-management skills by cardiovascular diseases patients, overcrowding at health facilities leading to poor management of cardiovascular diseases by nurses. | δ : time dedicated to cardiovascular diseases management  ε : lack of material, shortage of nurses | α : limited generalizability  β : to improve care and management of cardiovascular disease in rural populations, local  governments need to employ more skilled nurses whilst availing the necessary material resources |
| **First Author, Year - Location** | **Aim** | **Study Design** | **Disease** | **Sample** | **Results – Role or Knowledge of nurses** | **δ : Facilitators**  **ε : Barriers** | **α : Limitations**  **β : Perspectives** |
| ***Metabolic diseases*** | | | | | | | |
| Goodman, 1997 – South Africa | To audit staff knowledge, attitudes, and practices in the interest of improved public sector primary care for diabetics | Cross-sectional | Diabetes | 12 doctors, 23 nurses | Knowledge of chronic diabetic complications was adequate. There were gaps in knowledge of pathophysiology and of signs and symptoms of diabetic emergencies. Knowledge of appropriate care of patients with hypoglycemia was better than that of hyperglycemia. | δ : no identified  ε : lack of post basic training for nurses among diabetes | α : small sample  β : develop post-basic training in the nursing area |
| Koura, 2001 - Egypt | To study the role of primary health care in patient education for diabetes control in Alexandria by assessing the knowledge and perception of PHC providers concerning diabetes and its management, the knowledge and attitude of diabetic patients concerning self-management, measuring the degree of diabetic patients' satisfaction with the provided PHC services | Cross-sectional | Diabetes | 88 doctors - 104 nurses -560 diabetic patients | Nurses had some misconceptions and false beliefs (diabetes as a contagious disease, primarily caused by stress, low knowledge of risk factors), and had good knowledge of nutrition regarding diabetics’ patients. Patients had poor knowledge about diabetes and its management (85,7%) and negative attitude toward self-management (61,6%), only 23,6% were satisfied with the services provided by primary health care facilities: they were not satisfied with their education. Only 6,3% of patients attended health education sessions. | δ : primarily health care facilities for diabetes control exist  ε : commonly held myths and misconceptions, inappropriate practice regarding diabetes and its management | α : use of a non-validated questionnaire  β : need to promote diabetes education, continuous education, medical and nursing institution are invited to review curricula to strengthen knowledge, skills and attitudes essential for optimal diabetes care |
| Louwagie, 2002 - South Africa | To compare the clinical competencies and clinical management of nurses who obtained an "Advanced diploma in Health Assessment, diagnosis and treatment" with those who did not. | Cross-sectional | Diabetes | 51 nurses | Formal training was not associated with better care for diabetes (p=0.47). Other factors associated with more thorough care were years of experience in curative primary health care (p=0.006) and the presence of enrolled or assistant nurses at the clinic for diabetes (p=0.06). Fixed clinics generally performed better than mobile and satellite clinics. | δ : nurses identified as the cornerstone of chronic disease treatment in primary care settings, enrolled assistant nurses could be valuable  ε : diabetes management not standardized | α : study design and the analysis were based on medical records which were not always created for “visitors”.  β : improving teaching method for chronic disease |
| **First Author, Year - Location** | **Aim** | **Study Design** | **Disease** | **Sample** | **Results – Role or Knowledge of nurses** | **δ : Facilitators**  **ε : Barriers** | **α : Limitations**  **β : Perspectives** |
| Van de Sande, 2007 - South Africa | To investigate if there is a relationship between patients perceived diabetes education and their glycemic control in a primary health care clinic in South Africa. | Cross-sectional | Diabetes | 247 patients | Nurse led primary health care clinic with nurses specialized in diabetes. Treatment in this clinic was according a protocol based on the clinical practice recommendations of the American Diabetes Association.  90% patient received information about diabetes and 80% received information from a nurse. Patients who received information from a nurse had better fasting blood glucose levels. | δ : nurses trained, treatment based on protocol, favorable environment  ε : transports and weather problems | α : not validated questionnaire  β : patient education must be develop in nursing care |
| Gill, 2008 – South Africa | To set up and evaluate a nurse-led protocol and education-based system in rural Kwazulu Natal in South Africa for diabetes | Cross-sectional | Diabetes | 2 nurses  980 patients | After training, nurses with an algorithm could initiate diabetes treatment and education.  Baseline HbA1c was 11.1 ± 4.2% (n=284) and fell rapidly to 8.7 ± 2.6% (n=215) at 6 months and there was a slower but sustained fall to 7.9 ± 2.2% (n=197) at 18 months (p <0.001). Self-reported hypoglycemic rates did not significantly increase. | δ : nurses trained, use of algorithm  ε : shortage of transports | α : evaluation method, other outcome were needed  β : nurse led diabetes program could be introduced in rural africa |
| Mutea, 2008 - Kenya | To determine the extent of involvement of nurses in the management of diabetic patients in a Kenyan hospital | Qualitative | Diabetes | 15 nurses | Nurses identified diabetes as a chronic disease needing specific treatment and education with the possibility of complications. They recognize their professional implication in diabetes management. However, they perceived lack of time for regular blood, urine testing and basic teaching about medications and self-care. Had frustration with illiterate, poor patients and those who deny long-term illness. | δ : nurses trained and implicated in diabetes management, Kenyan diabetic association provides education and support  ε : nurse shortage, cost of care, lack of resource (medication and material), lack of organization | α : no identified  β : health literacy for patient is a key to improve outcome of diabetes. Need for clear engagement in this way |
| **First Author, Year - Location** | **Aim** | **Study Design** | **Disease** | **Sample** | **Results – Role or Knowledge of nurses** | **δ : Facilitators**  **ε : Barriers** | **α : Limitations**  **β : Perspectives** |
| Kengne, 2009 - Cameroon | To implement a protocol-driven primary nurse-led care for type 2 diabetes in rural and urban Cameroon | Prospective | Diabetes | 3 nurses  225 patients | A clinical management algorithm was designed from international guidelines to assist nurses’ diabetes evaluation and drug prescription (initiation and scaling up).  The difference in mean levels of fasting glucose between baseline and final visit was 1.6 mmol/L (p < 0.001). | δ : nurses training, use of an algorithm, nurse could prescribe, medical supervision, the program include referral  ε : lack of material, poor initial training | α : the study focused only on medium term outcome  β : nurses can deliver diabetes care, randomized trial are needed |
| Price, 2011 - South Africa | To report longer term outcome (4 years since initiation) from the Hlabisa Diabetes Project, to examine whether such improved control could be maintained | Cohort | Diabetes | 80 patients | Trained nurses used of validated algorithm to confirm diagnoses, initiate treatment, titrated treatment, trial of lifestyle modification and education.  HbA1c fell significantly to 8.1 at 6 months and 7.5 at 18 months. By 24 months, it had risen 8.4 and at 4 years post-intervention it was 9.7 (significantly lower than base- line, p = 0.015). | δ : regular training, use of algorithm  ε : education fatigue | α : high rate of loss of follow up  β : this program could be extended to other chronic disease |
| Matimba, 2016 - Zimbabwe | To establish the baseline prevalence of eye complications among diabetic patients at an outpatient diabetic clinic and provide an empirical framework to broaden the use of tele-ophthalmology in Zimbabwe in the future. | Cross-sectional | Diabetes eyes complications | 20 nurses  235 patients | Nurses were trained to tele-ophthalmology and on the use of the handheld non-mydriatic digital fundus imager  Patients were diagnosed for: non-macular diabetic retinopathy (11%), diabetic macular oedema (5%), cataract (5%) and glaucoma (6%). | δ : training  ε : need of ophthalmologists to review the images | α : technic was not compared with gold standard  β : nurses could be used to assess diabetic retinopathy with teleophthalmology and for providing patient education |
| **First Author, Year - Location** | **Aim** | **Study Design** | **Disease** | **Sample** | **Results – Role or Knowledge of nurses** | **δ : Facilitators**  **ε : Barriers** | **α : Limitations**  **β : Perspectives** |
| Essien, 2017 -Nigeria | To evaluate whether an intensive and systematic Diabetes self-management education program, using structured guidelines, improved glycemic control com-  pared to the existing ad hoc patient education | Randomized controlled trial | Diabetes | 118 | A nurse-led intensive educational program was created on international guidelines.  Intensive group participants had a mean six-month HbA1c (%) of 8.4 (95% CI: 8 to  8.9), compared to 10.2 (95% CI: 9.8 to 10.7) in control group (p < 0.0001). | δ : use of specialized nurses in education  ε : no identified | α : monocentric, some educative sessions were run by doctors, only six months follow-up  β : diabetes intensive education could be done by nurses in other low- and middle- income countries |
| Ndayisaba, 2017 - Rwanda | To describe how mentorship and enhanced supervision for  health care and quality improvement operate and evaluate  the quality of how mentorship and enhanced supervision for  health care and quality improvement supported diabetes care provided  in the context of decentralizing NCD services in three  rural districts of Rwanda. | Retrospective records analysis | Diabetes | 263 patients | A trained nurse used the protocol algorithms to make decisions for diagnosis and treatment or referral.  Of 263 observed nurse mentee-patient encounters, mentor and mentee agreed on diagnosis 94.4% of the time. Similarly, agreement levels were high for medication, laboratory exam, and  follow-up plans, at 86.3%, 87.1%, and 92.4%, respectively | δ : training, mentorship, use of checklists and algorithm  ε : no identified | α : data on clinical outcome of the patients were not extracted, potential desirability bias  β : mentorship is a key point to implement nurse led activities regarding NCDs management |
| Garanet, 2018 - Mali & Burkina faso | To explore educational concerns among diabetic’s patients | Cross-sectional | Diabetes | 17 nurses  61 doctors  1 dietetician | The median number of patients seen in consultation was 10 per week (range 1-100). The median age of experience in diabetes care for health professionals was 5 years (range: 1-25 years). Sixteen participants reported giving advice for cooking. Of the 62 that did not, the reasons given were mainly: non-competence to give such advice (60/62); lack of time (35/62) and useless counseling (5/62). For out-of-home meals, recommendations included: taking a meal to work (60/77); avoiding meals outside the home (30/77); avoid alcohol and oils at parties (10/77). | δ : no identified  ε : no identified | α : lack of patients’ data, small and specific sample  β : need for an harmonization of dietetics advises |
| **First Author, Year - Location** | **Aim** | **Study Design** | **Disease** | **Sample** | **Results – Role or Knowledge of nurses** | **δ : Facilitators**  **ε : Barriers** | **α : Limitations**  **β : Perspectives** |
| Kuguyo, 2020 - Zimbabwe | To understand behavioral, cultural, and religious factors including lack of knowledge about diabetes, poor drug adherence, lack of access to blood-glucose monitoring, and non-adherence to diet and exercise recommendations these factors provide a first step in developing appropriately tailored preventive interventions | Qualitative | Diabetes | 30 nurses | Four major themes were identified poor socio-economic status, poor self-care, cultural and religious factors, and health-system-  related factors. Lack of awareness results in poor health-seeking behavior, and use of unconventional treatment methods, which may increase Diabetic Foot risk among people living with diabetes, both diagnosed and undiagnosed. | δ : favorite prevention than treatment only, educate patient  ε : no identified | α : nurse’s centered  β : developpe tailored countrywide interventions |
| Bosun-Arije, 2020 - Nigeria | To examine healthcare delivery services influencing patient management and seek approaches to heighten optimization of patient health outcomes. | Qualitative | Diabetes | 17 nurses | Nurses suggested that a complex, multifaceted system constituted organizational factors influencing diabetes management in public hospitals across Lagos, Nigeria. Specific factors identified were levels of available information and knowledge, relationship, policy and decision-making management. These factors were, in turn, linked to political, infrastructural, health professional and the environments within which patients were given health services. | δ : high level of knowledge, favorable environment  ε : no identified | α : no triangulation, context-based information that might not be transferable and applicable  β : Provision of conducive clinic set-up and initiate flexible coordination of the diabetic clinic, preservation of professionalism and equity in clinical practice, organize workshops for health professionals in a public hospital setting across Nigeria and publish research on the outcome in hospitals’ newsletter for application to practice |
| **First Author, Year - Location** | **Aim** | **Study Design** | **Disease** | **Sample** | **Results – Role or Knowledge of nurses** | **δ : Facilitators**  **ε : Barriers** | **α : Limitations**  **β : Perspectives** |
| Hailu, 2021 - Ethiopia | To evaluate the potential of nurse-led, locally contextualized diabetes self-management education to modify psychosocial symptoms and HRQoL in a resource-limited setting | Before and after study | Diabetes | 220 patients | Nurses performed locally contextualized diabetes self-management education. Statistically, no significant differences were observed in depressive symptoms, stress, and HRQoL. Even though statistically not significant the proportion of participants who had eye examination at the endline was slightly greater in the intervention group. Intervention group participants reported that sessions be helpful for the management of stress and depressive symptom. | δ : training  ε : gaps in the adaptation of the education materials for a resource-limited setting, and modifications for low-literacy | α : high attrition bias, potential social desirability bias  β : adapted tool to local reality and need is a key component for effective patient education |
| O’Brien, 2020 - South Africa | To describe the experiences of diabetes nurse educators in relation to self-management of diabetes of persons living with type 2 diabetes | Qualitative | Diabetes | 5 nurses | Three themes were identified: diabetes nurse educators have clear perceptions  about the importance of self-management of Type 2 diabetes of persons living with Type 2 diabetes, there are factors that affect persons living with Type 2 diabetes with respect to self-management and there are ways in which professional nurses can assist persons in the self-management of their condition. | δ : training, regular patient education  ε : no identified | α : lack of input of non-specialized nurses  β : patient education performed by nurses could improve patients’ condition |
| **First Author, Year - Location** | **Aim** | **Study Design** | **Disease** | **Sample** | **Results – Role or Knowledge of nurses** | **δ : Facilitators**  **ε : Barriers** | **α : Limitations**  **β : Perspectives** |
| Asante*,* 2020 - Ghana | To evaluate the feasibility and effectiveness of a nurse-led mobile phone call intervention on glycemic management and adherence to self- management practices among patients with type 2 diabetes mellitus in Ghana | Randomized control trial | Diabetes | 60 patients | A mobile phone call intervention delivered by nurses in addition to care versus standard care was performed.  Mean baseline A1C was comparable between the intervention and control groups (9.54%, SD = 2.00% vs 9.07%, SD = 1.72%, p = 0.334). After 12 weeks, A1C was significantly lower in the intervention group compared to the control group (p = 0.004). No improvements in self-management were recorded in the control group. Intervention group significantly improve foot care practices. Participant recruitment and retention were 100% without any attrition. About 87% (n = 26) of the intervention group completed at least 70% (≥11) of the calls. At the end of the trial, participants who received the intervention rated their satisfaction as 89.3%. | δ : training, supervision, phone call intervention  ε : no identified | α : only type 2 diabetes with oral drug, lack of power to be able to identify other benefits to diabetes self-management, unvalidated instrument  β :A mobile phone follow-up call by nurses emphasizing adherence to self-management practices is feasible and can improve short- to medium-term glycemic management among patients with type 2 diabetes |
| Tamiru, 2023 - Ethiopia | To assess the effect of diabetes self-management education on self-care knowledge and behavior among adult people with type 2 diabetes attending the diabetic follow up clinic at Ilu Ababor and Buno Bedelle zone hospitals | Quasi-experimental | Diabetes | 321 patients | In the intervention group, nurses performed education on diabetes self-management every two weeks in addition to usual care.  Statistically significant higher mean score difference in self-care knowledge and self-care behavior after the delivery of the nurse-led diabetes self-management education was found in the interventional group compared to control. In the intervention group, the level of participant knowledge in the low range decreased from 62.7% to 20.6%, and the high range increased from 11.8% to 54%. | δ : program delivered by trained nurses  ε : transfer to other health institution | α : COVID 19 pandemic  β : nurse-led diabetes self-management education is an effective way to improve patient’s knowledge and self-care. |
| **First Author, Year - Location** | **Aim** | **Study Design** | **Disease** | **Sample** | **Results – Role or Knowledge of nurses** | **δ : Facilitators**  **ε : Barriers** | **α : Limitations**  **β : Perspectives** |
| Korsah, 2023 - Ghana | To explore the perception of nurses on the use of mobile phone SMS for managing diabetes in rural Ghana | Qualitative | Diabetes | 13 nurses | Participants believe SMS was useful in facilitating interaction between nurses, clients and family improving medication adherence and supporting blood glucose monitoring. The use of infographics was preferred to traditional SMS. Participants had limited knowledge of downloadable diabetic applications. Participants were willing to accept and use SMS for the management of diabetes mellitus. | δ : use of sms, infographics, alarms are favorable for diabetes medication adherence    ε : authorization by health local authorities, ability to manipulate phones | α : researcher presence, only nurses were studied  β : Mobile health seems to be a promising strategy for improving the care of diabetic patients in rural area |
| ***Neurological diseases*** | | | | | | | |
| Adamolekun, 1999 – Zimbabwe | To evaluate the effectiveness of primary health care nurses in the diagnosis and management of epilepsy as well as the impact of patient-information pamphlets on drug compliance and clinic attendance of patients with epilepsy | Prospective impact-evaluation study | Epilepsy | 31 nurses  400 patients | Nurses were able to diagnose epilepsy without false negative cases. There was an increase of 74% in cases in 6 months. They initiated phenobarbitone therapy in their health center without a medical referral. They had a positive impact on attendance.  The control (no pamphlet) group had a patient default rate of 56.3%, compared with 22.3% for the experimental group (p < 0.001).  There was no significant difference in the magnitude of change in seizure frequency and in the mean serum levels of phenobarbitone between groups. | δ : training in diagnosis and treatment of epilepsy (WHO guideline), could prescribe phenobarbitone, inexpensive  ε : no identified | α : short post-intervention follow up could underestimate the effect of patient information pamphlets  β : develop education of healthcare to manage epilepsy |
| **First Author, Year - Location** | **Aim** | **Study Design** | **Disease** | **Sample** | **Results – Role or Knowledge of nurses** | **δ : Facilitators**  **ε : Barriers** | **α : Limitations**  **β : Perspectives** |
| Adamolekun, 2000 - Zimbabwe | To evaluate the effectiveness and pattern of drug management of epilepsy by community health nurses who did not have any formal training in epilepsy management and did not have access to a treatment protocol | Cross-sectional | Epilepsy | 194 patients | Nurses done follow-up of epilepsy patient and monitoring of drug therapy. No change in therapy was necessary in 43%, an increase in drug dose was required in 29% of controlled consultations done by neurologists. Nurses appeared uncertain regarding change drug therapy, reduce polytherapy or stop drug therapy in clinically indicated situations. | δ : no identified  ε : no basic formation on epilepsy | α : no identified by authors  β : need to design programs for the training of community health nurses in the management of epilepsy |
| Kengne, 2008 - Cameroon | To set up and evaluate a nurse-led care for epilepsy at primary level in a rural health district in Cameroon | Prospective non-randomized interventional program | Epilepsy | 4 nurses  224 patients | After training, nurses were authorized to prescribe epilepsy treatment on an individual basis. During follow-up, there was a significant downward trend in the number of days per month with seizures in the overall cohort and among subgroups (p < 0.024) The program attracted over two times more new patients than those referred for the baseline survey. | δ : nurses were authorized to prescribe, training, supervision and availability of doctor if needed  ε : lack of material | α : short follow up period. absence of electroencephalographic evaluation  β : nurse-led protocol driven care for epilepsy is feasible and effective, randomized controlled study are needed |
| Rhodat, 2015 - Namibia | To determine the factors associated with knowledge of dysphagia amongst nurses at an intermediate hospital in Namibia | Cross-sectional | Stroke | 188 nurses | Nurses had a moderate knowledge of the signs, symptoms, and complications of dysphagia, but poor knowledge about its management. Training and experience in the care of dysphagia patients was a stronger predictor of knowledge than the initial qualification or years of experience as a nurse. | δ : training, experience  ε : no identified | α : convenience sampling, mono centric  β : specific training program on stroke related dysphagia need to be implement |
| **First Author, Year - Location** | **Aim** | **Study Design** | **Disease** | **Sample** | **Results – Role or Knowledge of nurses** | **δ : Facilitators**  **ε : Barriers** | **α : Limitations**  **β : Perspectives** |
| Kaddumukasa, 2019 - Uganda | To evaluate the feasibility, acceptability, and preliminary efficacy of an adopted novel  self-management intervention | Prospective uncontrolled pilot study | Epilepsy | 17 patients | A nurse led peer educator interactions. Self-management for people with epilepsy and a history of negative health events was associated with improved quality of life and reduced depression, stigma, and seizure frequency. | δ : The role of community leaders, local governments, and tackling stigma within communities would facilitate  implementation of these self-  management interventions  ε : lack of clear social support  mechanisms for people with epilepsy | α : small sample, selection bias (hospital based)  β : stigma is always a major problem for people with epilepsy |
| **First Author, Year - Location** | **Aim** | **Study Design** | **Disease** | **Sample** | **Results – Role or Knowledge of nurses** | **δ : Facilitators**  **ε : Barriers** | **α : Limitations**  **β : Perspectives** |
| Pierpoint, 2020 - South Africa | To explore how doctors and registered nurses, on initial clinical contact, identify and manage post-stroke dysphagia | Descriptive | Post stroke dysphagia | 21 nurses  4 doctors | 86% did not use a formal screening tool. Indicators screened informal included: presence of drooling (84%) or gag reflex (76%), level of alertness (80%) and spontaneous saliva swallow (80%). Participants neglected important indicators like voluntary cough and vocal quality. Management provided included head of bed elevation (96%), speech-language therapist referrals (92%), nasogastric tube insertions (88%), intravenous fluids (84%) and positional adjustments (76%). Alternative management included total parenteral nutrition (52%), syringe feeding (48%), swallow muscle strengthening exercises (56%) and swallow maneuvers (52%).  89% (16 of 18) of the registered nurses identified dysphagia | δ : no identified  ε : no identified | α : poor external validity, non-probability sampling, poor response rate  β : need to develop a South African dysphagia  screening tool |
| Knight, 2020 - South Africa | To describe nurses’ knowledge of stroke-related in the Eastern Cape, South Africa which may identify possible training needs and guide future interprofessional collaboration | Cross-sectional | Post stroke dysphagia | 130 nurses | The mean scores of correct responses for each section were: 8.7/13 (66.7%) for signs and symptoms, 4.7/10 (47.3%) for complications and 3.8/7 (54.2%) for management practices. Statistically, there were no differences between the levels of healthcare for the signs and symptoms section and the complications section. Regarding management of oropharyngeal dysphagia, secondary-level nurses demonstrated significantly better knowledge than primary-level and tertiary-level nurses (p = 0.010). | δ : no identified  ε : no basic training | α : monocentric, town setting  β : interdisciplinary  collaboration between nurses and speech–language therapists may improve nurses’ knowledge  in identification and management of stroke-related dysphagia |
| **First Author, Year - Location** | **Aim** | **Study Design** | **Disease** | **Sample** | **Results – Role or Knowledge of nurses** | **δ : Facilitators**  **ε : Barriers** | **α : Limitations**  **β : Perspectives** |
| ***Cancers*** | | | | | | | |
| Odusanya, 2001 - Nigeria | To assess nurses’ knowledge of breast cancer | Cross-sectional | Breast Cancer | 280 nurses | Knowledge about symptoms, methods of diagnosis and self-breast examination was generally very good. 30% had had a clinical breast examination and 8% a mammogram within the past three years. Use of cancer screening methods was significantly associated with knowledge of the subject. 28% did not know how to estimate the risk of cancer and 61% believed they were not at risk. | δ : no identified  ε : lack of knowledge on risk factors of breast cancer | α : no identified by authors  β : need to continuous education in breast cancer, especially regarding risk factors and breast cancer risk examination |
| Ayinde, 2003 - Nigeria | To find out the level of awareness of female health workers about cervical cancer and the level of utilization of preventive measures | Cross-sectional | Cervical cancer | 90 nurses  25 doctors  70 hospital maids | Knowledge about the condition was high among doctors, surprisingly inadequate among nurses. 93.2% of respondents have never had Pap smears performed independently of respondent’s profession, marital status, or hospital. | δ : no identified  ε : lack of training | α : no identified by authors  β : need to develop knowledge and prevention of cervical cancer |
| Anya, 2005 - Nigeria | To assess knowledge attitude towards cervical cancer prevention detection in female health workers | Cross-sectional | Cervical cancer | 85 nurses  75 others health workers | 91,7% had hear of cervical cancer, 22,2 % could not list any risk factor. For nurses 32,9% believed it was potentially curable and 71,1% that it could be prevented, 59,2% thank that pap smear screening is for prevention, 5,3% had pap smear. | δ : Nigeria had taken pap smear as an important public health problem.  High level of knowledge  ε : poor availability of pap smear screening | α : no identified by authors  β : Better promote positive attitude toward cervical cancer prevention |
| **First Author, Year - Location** | **Aim** | **Study Design** | **Disease** | **Sample** | **Results – Role or Knowledge of nurses** | **δ : Facilitators**  **ε : Barriers** | **α : Limitations**  **β : Perspectives** |
| Sangwa-Lugoma, 2006 - Democratic Republic of Congo | To assess the feasibility and performance of VIA and VILI for cervical cancer  screening in a primary healthcare setting in Kinshasa, Congo | Cross-sectional | Cervical cancer | 6 nurses  1571 women | Nurses may learn to master the different steps of the screening activities, including subject recruitment, public health education, performance of clinical examination and specimen collection procedures. | δ : limited cost, 10-days training for nurses (teaching and practical sessions) and supervision by gynecologist  ε : no structured screening existed before the study | α : no identified by authors  β : Train more nurses to pap smear screening could be a solution for early identification of cervix cancer |
| Udigwe, 2006 - Nigeria | To determine the knowledge and practice of cervical cancer screening among female nurses with a view to sensitizing them as a first step towards increasing screening uptake in community | Cross-sectional | Cervical cancer | 144 nurses | 87% of nurses were aware of the existence of screening services. Only 42 % got their knowledge from formal lectures. 5,7% had ever undergone a pap smear screening, 37,1 had no reason for not screening, 15% were afraid about the possible outcome.  Obstacles to uptake of cervical screening has been conflicting health beliefs, cultural taboos, interference with sexual relationship and denial driven by fear of cancer. | δ : no identified  ε : no structured screening existed | α : study design  β : Further education on pap smear is needed. Nurse could play a major role in screening services |
| Ibrahim, 2009 - Nigeria | To assess knowledge of breast cancer risk factors, beliefs about treatment and practice of screening methods among female healthcare professionals | Cross-sectional | Breast cancer | 141 nurses  45 doctors  13 laboratory scientists  4 pharmacists  4 | Mean knowledge score for nurses was 35% compared to 74% for doctors (p<0.01). 43% among nurses possessed poor knowledge and only 2 (1%) had excellent knowledge. | δ : no identified  ε : no identified | α : high proportion of nurses, small proportion of study participants delayed return of the questionnaires for several hours  β : Frequent continuing medical education programs on breast cancer at institutional level is desirable. Greater emphasis needs to be placed on breast cancer in nursing curricula |
| **First Author, Year - Location** | **Aim** | **Study Design** | **Disease** | **Sample** | **Results – Role or Knowledge of nurses** | **δ : Facilitators**  **ε : Barriers** | **α : Limitations**  **β : Perspectives** |
| Akhigbe, 2009 - Nigeria | To assess the awareness of female health workers about risk factors and screening methods for early detection of breast cancer. | Cross-sectional | Breast cancer | 254 nurses  102 doctors  37 radiographers, laboratory, pharmacist | The doctors had a significantly higher mean knowledge score, 2.32 ± 0.85 than the other categories of health workers (p < 0.0001), nurses 1,34+/-0,83. The doctors (39.2%) had significantly higher knowledge of the risk factors than the nurses (9.8%).  55.0% had very poor knowledge about the risk factors for breast cancer. The doctors had a significantly higher mean knowledge score of 4.92 ± 1.77, compared with other categories of health workers (p < 0.0001), nurses 3.33 ± 2.32. | δ : no identified  ε : no formation on breast cancer in curricula | α : no standardized questionnaire for breast cancer knowledge  β : integrate breast cancer knowledge in curricula |
| Awodele, 2009 - Nigeria | To assess the knowledge, attitude, and practice of breast cancer screening among nurses | Cross-sectional | Breast cancer | 183 nurses | 100% rate of awareness of breast cancer although 32% of the respondents knew not that breast cancer could be inherited. 76% of their information about breast cancer was from health professionals. 96% knew self-breast examination while 41% knew clinical breast examination as screening method. | δ : no identified  ε : no identified | α : study design  β : need for educational intervention to enhance knowledge of the risk factors and the need for clinical breast examination |
| Awodele, 2011 - Nigeria | To investigate the knowledge, attitude, and practice of nurses in Lagos University Teaching Hospital towards cervical cancer screening | Cross-sectional | Cervical cancer | 200 nurses | 99% of the respondents were aware of cervical cancer and that 92% of the respondents were also aware of the causative organism of cervical cancer. Major sources of information were through electronic media (43.9%) and health professionals (37.4%). 91% of nurses were aware of Pap smear as one of the screening techniques of cervical cancer and 89% had good attitudes towards. | δ : no identified  ε : socio economic cost of pap smear | α : study design  β : regular trainings for nurses are needed because they should give health education to women about cervical cancer |
| **First Author, Year - Location** | **Aim** | **Study Design** | **Disease** | **Sample** | **Results – Role or Knowledge of nurses** | **δ : Facilitators**  **ε : Barriers** | **α : Limitations**  **β : Perspectives** |
| Ghanem, 2011 - Morocco | To assess knowledge of breast cancer risk factors, beliefs about treatment and practice of screening methods among female healthcare professionals | Cross-sectional | Breast cancer | 92 nurses  44 doctors | Nurses had an unsatisfactory knowledge with a mean score of 43%. 56.5% among nurses possessed poor knowledge and only 1(1%) had excellent knowledge. 40% among nurses believed that breast cancer cannot disappear following prayer without treatment. | δ : no identified  ε : wrong cultural and religious limitation | α : no identified  β : Programs to educate religious leaders and alternative medical practitioners about breast cancer  should be encouraged |
| Urasa, 2011 - Tanzania | To determine nurses’ awareness of cervical cancer and their own screening practices at a hospital in Tanzania | Cross-sectional | Cervical cancer | 137 nurses | Less than half of the nurses had adequate knowledge regarding cervical cancer. There was a significant association between knowledge levels of causes of cervical cancer and transmission of HPV and age. Knowledge was more adequate among the young nurses (p = 0.027). Registered nurses had more adequate knowledge than enrolled nurses (p = 0.006). The majority did not know screening intervals, and a few were aware of HPV vaccine. Most nurses (84.6%) had never had a Pap smear examination. | δ : mediatization of cervical cancer  ε : bad knowledge in nursing school | α : monocentric study  β : improve continuing medical education, create cervical cancer prevention policies, improve nursing school curricula |
| Bello, 2011 - Nigeria | To evaluate the relationship between knowledge and practice of breast cancer screening in nurses and lay woman | Cross-sectional | Breast cancer | 74 nurses | Nurses had good knowledge of breast cancer, especially on signs and symptoms, they were aware of screening modalities. | δ : no identified  ε : no identified | α : no identified  β : improve breast cancer screening |
| **First Author, Year - Location** | **Aim** | **Study Design** | **Disease** | **Sample** | **Results – Role or Knowledge of nurses** | **δ : Facilitators**  **ε : Barriers** | **α : Limitations**  **β : Perspectives** |
| Arulogun, 2012 - Nigeria | To assess the perception and utilization of cervical cancer screening services among female nurses in University College Hospital, Ibadan, Nigeria. | Cross-sectional | Cervical cancer | 503 nurses | Mean knowledge scores by cadre were Assistant Directors (26.7 ± 1.5), Chief Nursing Officers (23.4 ± 2.3) and Staff Nurses (21.7 ± 5.3) (p<0.05). 88.0% correctly perceived cervical cancer to be preventable and 82.0% believed that screening should be carried out as soon as sexual intercourse starts. Staff Nurses were four times less likely to utilize cervical screening services than the Assistant Directors of Nursing (OR 0.23, CI 0.117-0.442). | δ : no identified  ε : no identified | α : monocentric, focusing only on woman  β : need for cervical cancer screening education programs to be carried out among health professionals at all levels especially among nurses |
| Rwamugira, 2012 - South Africa | To develop an intervention focusing on the prevention and detection of oral cancer and pilot test the intervention in a semi-urban resource-poor community in Tshwane | Interventional pilot study | Oral cancer | 1320 patients | A flyer was used to screen and educate the global population, patients referred to the nurse for examination. 65 patients were diagnosed. The flyer improves knowledge and awareness of population. | δ : the screening was free, the flyer was translated in local languages  ε : lack of effective screening strategies to attract people | α : purposive sampling in a chosen area  β : Nurse could be used to screen oral cancer |
| Moon, 2012 - Mozambique | To describe key challenges of rolling out a VIA-based cervical cancer screening program in a rural, extremely resource-limited setting | Cross-sectional | Cervical cancer | 4651 patients | After training, maternal child health nurses effectively screened woman for cervical cancer in HIV center. VIA was judged positive for squamous intraepithelial lesions in 8% (n=380) of the women 4% (n=16) had lesions (0.3% of 4651 total screened) requiring referral to hospital. Of women screened, 2714 (58%) either had knowledge of their HIV status prior to VIA or were subsequently sent for HIV testing, of which 583 (21%) were HIV positive. | δ : use of already existing services (HIV center)  ε : lack of effective physical infrastructure, human resources | α : absence of cytopathology and quality assurance of screening  β : Screening and clinical services were successfully provided by nurses on a large scale for the first time in rural clinics, nurses led cervical cancer screening using VIA is feasible in resource-constrained rural Africa |
| **First Author, Year - Location** | **Aim** | **Study Design** | **Disease** | **Sample** | **Results – Role or Knowledge of nurses** | **δ : Facilitators**  **ε : Barriers** | **α : Limitations**  **β : Perspectives** |
| Wamai, 2013 - Cameroon | To investigate the knowledge and awareness of HPV, primary cause of cervical cancer and HPV vaccine among nurses working at four Cameroon Baptist Convention  Health Services facilities, and to explore what factors influence nurses’ willingness to inform and recommend HPV vaccine to adolescents and parents attending clinics | Cross-sectional | Cervical cancer | 76 nurses | There were moderately low levels of knowledge about HPV infection and prevention of cervical cancer, but a moderately high level of knowledge about HPV vaccine. Although 90.8% acknowledged that cervical cancer is directly linked to HPV infection, nearly 32% failed to identify it as a sexually transmitted infection, 43.4% believed it is an uncommon infection. Willingness to recommend the HPV vaccine was moderate, with 69.7% intentionally initiating discussions with patients about the subject. The most important factors considered when deciding to recommend the vaccine included effectiveness (56.6%) and side effects/safety (11.8%). Cost was less of a concern (6.6%), likely due to the availability of donated vaccine. | δ : no identified  ε : no identified | α : low response rate, small sample size  β : improve education among cervical cancer |
| Mwanahamuntu*.*, 2013 - Zambia | To evaluate the utilization of screening services by Zambian women stratified by HIV serostatus and examine the trends and factors associated with screening-test positivity over the program scale-up period | Retrospective records analysis | Cervical cancer | 17 nurses  56,247 women | Trained female Zambian nurses perform screening of women with VIA aided by digital camera enhancement of the cervix, cryotherapy was offered for eligible woman.  The proportion of women  accessing these services who were HIV-seropositive declined from 54% to 23% between 2006–2010. After adjusting for  demographic and sexual/reproductive factors, HIV-seropositive women were more likely (Odds ratio 2.62, 95% CI 2.49, 2.76) to screen VIA-positive than HIV-seronegative women. | δ : training for nurses for cervical cancer screening, peer training  ε : no identified | α : study design  β : use of HIV clinics to implement chronic disease screening is favorable |
| **First Author, Year - Location** | **Aim** | **Study Design** | **Disease** | **Sample** | **Results – Role or Knowledge of nurses** | **δ : Facilitators**  **ε : Barriers** | **α : Limitations**  **β : Perspectives** |
| Manga, 2015 - Cameroon | To evaluate interobserver agreement of digital cervicography results, compared digital cervicography with histopathologic results, and examined interobserver agreement among screening methods | Cross-sectional | Cervical cancer | 540 patients | Agreement between nurses was moderate [K = 0.54; 95% confidence interval (CI), 0.47–0.61], agreement between the first nurse and the gynecologist was fair (K= 0.37; 95% CI, 0.29– 0.44), agreement between the second nurse and the gynecologist was moderate (K= 0.45; 95% CI, 0.37–0.53) with no statistically significant difference between observers | δ : training  ε : no identified | α : unblinded histopathologist  β : Nurses using digital cervicography could be an option for primary cervical cancer screening in low- and middle-income countries |
| Firnhaber, 2015 - South Africa | To determine whether a quality assurance program using digital cervicography improved the per-  formance of a visual inspection with acetic acid (VIA) to detect cervical intraepithelial neoplasia grade 2 or worse (CIN 2+) in HIV-infected women in Johannesburg, South Africa | Cross-sectional | Cervical cancer | 1202 women | After specific training, nurses performed VIA of cervix, VIA were reviewed by a specialist.  Positive VIA results were found in 45%. The sensitivity of VIA to predict CIN 2+ was improved from 65% to 75% (p = .001) with the addition of digital cervicography and specialist review. There was no statistical difference in the sensitivity of the VIA readings when comparing between the nurses and physicians. | δ : training, low-cost procedure  ε : no identified | α : no identified  β : quality assurance program improves sensitivity of VIA performed by nurses |
| DeGregorio, 2017 - Cameroon | To document the strategies that have led to successful implementation of a nurse-led cervical cancer  screening program using VIA-Digital Cervicography | Restrospective | Cervical cancer | 46,048 patients | After training nurses performed cervical cancer screening | δ : training, appropriate culturally sensitization, free service  ε : lack of access to screening and treatment, shortage in health care | α : limitations related to VIA screening especially in older woman  β : fee-for-service model for VIA digital cervicography is feasible |
| **First Author, Year - Location** | **Aim** | **Study Design** | **Disease** | **Sample** | **Results – Role or Knowledge of nurses** | **δ : Facilitators**  **ε : Barriers** | **α : Limitations**  **β : Perspectives** |
| Asgary, 2016 - Ghana | To evaluate the feasibility and limited efficacy of a smartphone-based training of community health nurses in VIA of cervix | Cross-sectional | Cervical cancer | 15 nurses  169 women | After training nurses performed VIA.  The total agreement rate between all VIA  diagnoses made by all nurses and the expert reviewer was 95%. The average rate of agreement between each nurse and the expert reviewer was 89.6% (Standard Deviation (SD)=12.8). The agreement rates for positive and negative cases were 61.5% and 98.0%, respectively. Cohen's kappa statistic was 0.67 (95% CI; 0.45-0.88). Around 7.7% of women tested VIA positive received cryotherapy or further services. | δ : training  ε : no identified | α : no comparison groups  β : nurses has the potential to improve cervical cancer screening coverage in  Ghana |
| Dickerson, 2017 - South Africa | To assess the feasibility, primarily defined by knowledge acquisition, perceived usefulness, and provider self-efficacy, of a breast ultrasound  training program for nonphysician providers. | Pilot study | Breast cancer | 7 nurses  5 lay workers | The pre- to post-test averages improved by 68% in total and in four competencies: foundational knowledge, descriptive categories, benign vs malignant, and lesion identification.  On the postsurvey, providers expressed that ultrasound could significantly influence breast cancer detection, treatment, and survival in their community and endorsed moderate confidence in their scanning and interpreting abilities. | δ : 3-weeks complete training, low cost, maintenance of radiologist supervision  ε : low confidence in interpreting abilities for nurses | α : small sample, language for training was English  β : ultrasound could become a viable downstaging tool in other limited-resource settings where mammography is unavailable |
| Pace, 2018 - Rwanda | To assess the impact of the training intervention in breast cancer detection on knowledge and skills among health center nurses and community health workers | Cross-sectional | Breast cancer | 126 nurses  1026 community health workers | Nurses’ written test scores improved after the trainings from 73.9% to 91.3% (p < 0.001) sustained 3 months after the trainings.  On checklists that assessed skills, nurses’ median percentage of actions performed correctly was 24% before the training and 88% after and during mentorship % (p < 0.001). | δ : adequate  training, mentorship, established care delivery and referral systems  ε : coordination / communication | α : limited generalizability, not same baseline for carers  β : Nurses can play a critical role in earlier detection of breast cancer |
| **First Author, Year - Location** | **Aim** | **Study Design** | **Disease** | **Sample** | **Results – Role or Knowledge of nurses** | **δ : Facilitators**  **ε : Barriers** | **α : Limitations**  **β : Perspectives** |
| Williams, 2018 - Ghana | To investigate the awareness of cervical cancer, and knowledge of cervical cancer risk factors and methods of prevention among nurses in Ghana | Qualitative | Cervical cancer | 42 nurses | Awareness of cervical cancer was very high among the nurses. However, most of the participants held negative perceptions about cervical cancer and lacked knowledge about cervical cancer risk factors and prevention. There is stigma with cervical cancer. | δ : no identified  ε : stigma | α : purposive sample, time constrained interviews  β : nee to target the population for evidence-based intervention |
| Dareng, 2018 - Nigeria | To evaluate the secular trend in interobserver agreement between nurse providers and a gynecologist/ colposcopist over a five-year period | Cross-sectional | Cervical cancer | 70 nurses  4,961 patients | After theorical and practical training, nurses performed VIA of cervix.  Overall agreement was 0.89 at Site D, 0.78 and 0.73 at Sites A and C respectively, 0.50 for Site E and 0.34 for Site C. The number of trainings attended by nurse providers (β = 0.47, 95% CI:0.02–0.93, p = 0.04), high level of engagement by site gynecologists (β = 0.11, 95% CI:0.01–0.21, p = 0.04) were associated with increased agreement; while increasing distance from the coordinating site (β = -0.47, 95% CI:-0.92–0.02, p = 0.04) was associated with decreased agreement. There were no associations between number of years screening clinics were operational,  cumulative experience of nurse providers and agreement. | δ : regular onsite supervision, more formal VIA training sessions  and proximity to the cervical cancer screening coordinating site  ε : no identified | α : gynecologist was not blinded to nurse diagnostic  β : could help to select appropriate cervical cancer screening strategies for their populations |
| **First Author, Year - Location** | **Aim** | **Study Design** | **Disease** | **Sample** | **Results – Role or Knowledge of nurses** | **δ : Facilitators**  **ε : Barriers** | **α : Limitations**  **β : Perspectives** |
| Ndikom, 2019 - Nigeria | To evaluate the effects of an educational intervention on nurses’ knowledge and attitude towards providing cervical cancer screening information | Quasi-experimental | Cervical cancer | 133 nurses | Nurses were assigned to the educational intervention group or control. The intervention package for the program was adapted from Nurses’ Training Manual on Cervical Cancer Control by the WHO.  Knowledge scores for the intervention group (11.8 ± 3.3) and control group (11.7 ± 3.3) were comparable at baseline (p = 0.901) but was significantly higher among nurses in the intervention group (14.63 ± 3.12) than control group (12.7 ± 3.5) at 6 months post intervention (p = 0.01). More nurses in the intervention group had high level of knowledge than control group. There was a significant association between knowledge and attitude towards providing cervical cancer screening information (p < 0.001). | δ : use of WHO training tool  ε : no identified | α : self-administered questionnaire  β : need for observational studies evaluating in real life nurses’ knowledge and attitude towards providing cervical cancer screening |
| Asgary, 2019 - Ghana | To explore acceptability and feasibility of smartphone-based training of low-level to mid-level health professionals in cervical cancer screening using VIA cervicography | Qualitative | Cervical cancer | 15 nurses | Smartphone-based training and mentorship was seen as important for further developing diagnostic and management skills. Cervical imaging helped with patient communication and education. None of the patients had prior screening, and they accepted smartphone-based VIA. Nurses addressed lack of knowledge and misperceptions using existing community relationships. Patients had decision-making autonomy regarding screening, but negative views and stigma were present. The majority felt that screening strategies were acceptable and effective. | δ : training, mentorship  ε : no identified | α : small sample  β : large scale with smartphone-based technology and quantitative design are needed |
| **First Author, Year - Location** | **Aim** | **Study Design** | **Disease** | **Sample** | **Results – Role or Knowledge of nurses** | **δ : Facilitators**  **ε : Barriers** | **α : Limitations**  **β : Perspectives** |
| Pruitt, 2020 - Nigeria | To assess the breast cancer knowledge level of health care providers in Southwestern Nigeria | Cross-sectional | Breast cancer | 349 nurses  133 doctors  88 pharmacists  113 students | Median knowledge score was 31 out of 56 (interquartile range [IQR], 24-36) and ranged from 0-47 points. The differences in median scores between occupational groups were statistically significant (P < .001). Primary health center nurses and health care workers (median, 27; IQR, 21-31) scored significantly lower than other types of health care workers (median, 32; IQR, 26-38) on our knowledge assessment. Hospital-based providers (physicians, nurses, and pharmacists) had higher knowledge scores (median, 36; IQR, 32-40) than non–hospital-based providers (median, 26; IQR, 20-31). | δ : no identified  ε : no identified | α : limited geographic area, no validated assessment tool  β : Need to improve nurses knowledge on breast cancer |
| Domgue, 2020 - Cameroon | To examine the feasibility of WHO guidelines for cervical cancer prevention recommend a screen-and-treat strategy with high-risk HPV testing implementation in rural Cameroon | Implementation study | Cervical cancer | 1270 patients | Nurses performed a screen-and-treat strategy of cervical cancer. 196(15.4%) were HPV-positive of whom185(94.4%) were examined,16(8.6%) were VIA/VILI-positive,8(4.3%) were VIA/VILI-inadequate, one (0.5%) was VIA/VILI-uncertain and 161(87.0%) were treated with thermal ablation. | δ : proximity service, nurse training  ε : no identified | α : no identified  β : The use of local staff to liaise between the community and higher-level health care providers is essential to achieve high coverage and compliance in these rural community |
| **First Author, Year - Location** | **Aim** | **Study Design** | **Disease** | **Sample** | **Results – Role or Knowledge of nurses** | **δ : Facilitators**  **ε : Barriers** | **α : Limitations**  **β : Perspectives** |
| O’Neil, 2021 - Eswatini | To evaluate if a breast cancer early-detection program in Eswatini improve HIV clinic nurses’ breast evaluation skills and improve  evaluation of their patients’ breast symptoms | Prospective pilot study | Breast cancer | 44 nurses  9,502 patients | Following a 2-day training, nurses demonstrated significantly improved breast health knowledge and clinical breast ex-  amination skills. Those improvements were durable as well, with practically no deterioration at 90 days after training. | δ : training based on already existent program with positive results  ε : costs of transportation or care, work related time constraints, not convinced of the need for further breast evaluation, fear of a breast cancer diagnosis, physical distance to breast specialist patient, covid 19 precautions | α : plausible desirability bias  β : successful efforts to decrease the stage of breast cancer diagnosis in resource-limited health systems will require both effective detection  strategies and accessible services for timely diagnosis |
| **First Author, Year - Location** | **Aim** | **Study Design** | **Disease** | **Sample** | **Results – Role or Knowledge of nurses** | **δ : Facilitators**  **ε : Barriers** | **α : Limitations**  **β : Perspectives** |
| Mkhonta, 2021 - Swaziland | To explore the barriers to cervical cancer screening among nurses | Qualitative | Cervical cancer | 15 nurses | Four themes emerged which were  deterrents to cervical cancer screening. These were perceived client barriers (patient's fear, being screened by a male nurse, absence of the preferred nurse, lack of knowledge, the asymptomatic nature of cervical cancer, long walking distance to the facility, cultural beliefs,), health care system related barriers (accessibility and availability of services Shortage of equipment, Poor supervision from the cervical cancer program, cost of the screening test, no means for patient follow up, increased waiting time, busy working hours, not effective booking system, poor laboratory results system), nurse related barriers (shortage of staff, the gender of the nurse, shortage of trained nurses, a nurse who is a resident of the community, lack of support from colleagues) and the nature of  the procedure. | δ : culturally  ε : lack of material, shortage of nurses, patients’ fears | α : potential recall and desirability bias  β : extent health education to improve cervical cancer screening |
| Obol, 2021 - Uganda | To assess knowledge, attitudes, and practice of cervical cancer prevention among health workers employed in rural health centers in the Acholi sub-region of Northern Uganda | Cross-sectional | Cervical cancer | 153 nurses among 286 participants | Participants had adequate knowledge of cervical cancer. 187 (66%) participants had positive attitudes. Participants who indicated not to have ever received training on cervical cancer screening were less likely to have adequate knowledge (AOR = 0.39, 95% CI 0.21–0.71). | δ : training  ε : no identified | α : potential recall bias,  social desirability bias, small sample  β : government should develop and disseminate guidelines for cervical cancer prevention to rural health workers to promote standardized cervical cancer prevention activities |
| **First Author, Year - Location** | **Aim** | **Study Design** | **Disease** | **Sample** | **Results – Role or Knowledge of nurses** | **δ : Facilitators**  **ε : Barriers** | **α : Limitations**  **β : Perspectives** |
| Mango, 2022 –  Nigeria | To assess the iBreastExam performance compared with clinical breast examination for breast lesion detection in high risk and symptomatic Nigerian women, using mammography and ultrasound as the reference standard | Prospective | Breast cancer | 4 nurses  424 participants | After training, nurses provided breast examination with iBreastExam device. iBreastExam done by nurses showed significantly better sensitivity than clinical breast examination (63%, 95% CI 57–69 vs 31%, 25–37; p<0·0001), and clinical breast examination showed significantly better specificity (94%, 90–97 vs 59%, 52–66; p<0·0001) | δ : training by physicians  ε : potential cost | α : self-selected population, comparison with ultrasound that is not the gold standard  β : iBreastExam done by nurses might provide a high sensitivity breast evaluation tool |
| Taj, 2022 – Kenya | To assess knowledge improvement and retention among oncology  nursing staff immediately after 12-week of computer led eLearn-  ing sessions and at 3-month intervals up to a period of 12 months across four hospitals in Kenya and Tanzania | Pre- and posttest inter-  vention | Cancer | 21 nurses | Nurses received an eLearning intervention on cancer biology, epidemiology, pharmacology, safe chemotherapy administration, side effect management, and patient education.  There was an improvement in knowledge scores for 12 participants (p<0.05). The mean pre- and posttest results found significant differences across 10 sessions individually and cumulatively (p<0 .01).  There was no difference in knowledge retention across 9 months. | δ : South-North collaboration, use of advanced practice nursing to identify knowledge gaps.  ε : eLearning not culturally adapted | α : small sample  β : need to develop context specific programs |
| Getachew, 2022 - Ethiopia | To determine the efficacy of a trained breast nurse intervention on improving endocrine therapy self-reported adherence compared to control. | Cluster randomized study | Breast cancer | 21 nurses  162 participants | In the intervention group, trained nurses delivered education, literacy material, empathetic counselling, phone call reminders, and monitoring of medication refill at the intervention hospitals.  Control group consisted in usual care.  Adherence at intervention sites was 70% compared with 44.8% in the control sites (p = 0.03) at 12 months. Persistence to therapy was found to be 91.2% in the intervention and 77.8% in the control sites during the one-year period (p = 0.01) | δ : Nurses were trained on pathophysiology, communication, how to give medication remember call.  ε : access to medication | α : inclusion of patients with a prior history of endocrine intake, low participation of patients during adherence measurement at 12 months  β : breast nurses can improve cost-effective endocrine therapy adherence, task-sharing could overcome the shortage of oncologist and distances to central cancer center |
| **First Author, Year - Location** | **Aim** | **Study Design** | **Disease** | **Sample** | **Results – Role or Knowledge of nurses** | **δ : Facilitators**  **ε : Barriers** | **α : Limitations**  **β : Perspectives** |
| Chitha, 2023 – South Africa | To ascertain the knowledge, attitudes, practices and barriers of cervical cancer screening among nurses of selected rural hospitals in South Africa | Cross-sectional | Cervical cancer | 119 nurses | 15.1% (18/119) of participants were assessed as having obtained a good knowledge score of ≥ 65%. The majority of these (16/18, 88.9%) were professional nurses. Cervical cancer was deemed to be a disease of public health importance by 74.0% (88/119). However, only 27.7% (33/119) performed cervical cancer screening. Most of the participants (116/119, 97.5%) had an interest of attending more cervical cancer training. | δ : Nurses are key to the goals of improving the prog- nosis of cervical cancer survivors  ε : poor knowledge on health policy among cervical cancer, nurse curriculum, | α : small sample, use of questionnaire which could question the probity  β : Need to improve nurses knowledge on cervical cancer |
| ***Respiratory diseases*** | | | | | | | |
| Kengne, 2008 - Cameroon | To implement nurse-led care for asthma in rural Cameroon | Prospective interventional study | Asthma | 28 nurses  87 patients | After training, a stepped approach for the nurse management of asthma and algorithm were implemented in pilot’s clinic.  There was a significant downward trend in the number of days/months with attacks with the duration of follow-up, and at the last visit most patients had improved compared with the initial visit. | δ : Nurse were trained, medical supervision, regular evaluation, and training by doctor (One per month), refresh course a year after the initial training, use of algorithm  ε : no identified | α : short median duration follow-up, inability to accurately assess the compliance to medication, absence of respiratory function test  β : In rural settings nurse-led clinics represent an option to improve access to care for chronic diseases. |
|  |  |  |  |  |  |  |  |
| ***Psychiatric diseases*** | | | | | | | |
| Kgosidintsi, 1996 – Botswana | To identify and describe the role of the psychiatric/community mental health nurse in the context of primary health care | Qualitative | Schizophrenia | 9 community mental health nurses | 3 main functions: mental status assessments of patients, prescription, and administration of treatment to patients. It also includes supporting groups for families. | δ : governmental initiative  ε : costs and lack of transport | α : small sample  β : need to develop structures for individual and community mental care |
| **First Author, Year - Location** | **Aim** | **Study Design** | **Disease** | **Sample** | **Results – Role or Knowledge of nurses** | **δ : Facilitators**  **ε : Barriers** | **α : Limitations**  **β : Perspectives** |
| Ndetei, 2011 - Kenya | To determine the knowledge, attitudes, and beliefs about mental illness among staff in general hospitals | Cross-sectional | Mental illness | 327 nurses  124 doctors | There are gaps in knowledge on mental illness, but nurses reported that they felt responsible for following up common types of psychological disorders. Mental illness’s stigmatization is an important issue to address. | δ : no identified  ε : no identified | α : no identified  β : develop continuing medical education on mental illness |
| Adams, 2012 - Tanzania | To adapt a model of nurse led depression management an HIV clinic in Tanzania | Feasibility | Depression | 1 nurse  21 patients | A nurse was trained to use instruments to diagnose depression and the guideline-concordant depression treatment algorithm.  The nurse correctly identified all algorithm-indicated antidepressant recommendations. | δ : training, supervision, use of algorithm  ε : medication dosing and supply | α : small sample size and lack of a control arm  β : systematic screening of depression will be integrated and run by nurses |
| Chetty, 2013 - South Africa | To determine the effectiveness of a nurse-facilitated-cognitive-group (NFCG) intervention as an adjunct to antidepressant medication, in mild to moderately, depressed women | Quasi-experimental | Depression | 30 patients | Patients were randomly assigned to an intervention nurse-facilitated-cognitive-group or a control group. After 12 weeks the Beck Depression Index scores for the intervention group decreased (p<0.001) compared to control group. There was no improvement in the control group (p=0.597). | δ : training  ε : no identified | α : small sample size, one gender analyzed  β : nurse could help to answer the shortage of psychiatric specialists |
| Alonso, 2014 - Sierra Leone | To describe the results of a free outpatient mental health programme delivered by non-specialist health workers in Makeni,  Sierra Leone between July 2008 and May 2012. | Cross-sectional | Mental illness | 2 nurses  554 patients | Nurses were trained to identify and manage mental disorders.  417 patients were diagnosed suffering from psychotic disorders (n=295, 53.7%), maniac episodes (n=69,  12.5%), depressive episodes (n=53, 9.6%), drug use disorders (n=182, 33.1%), dementia (n=30, 5.4%), mental disorders due to medical  conditions (n=39, 7.1%), and developmental disorders (n=46, 8.3%). 417 patients received pharmacological therapy and 70.7%  were rated as much or very much improved. Of those who could not be offered medication, 93.4% dropped out of the program after the first visit. | δ : training  ε : treatment gap | α : the study was not designed as “research”, the training was specifically design for Sierra Leone and before the WHO guidelines implementation, need for objective measures of the quality and quantity of supervision required  β : trained primary nurses can deliver safe and effective treatment for mental disorders |
| **First Author, Year - Location** | **Aim** | **Study Design** | **Disease** | **Sample** | **Results – Role or Knowledge of nurses** | **δ : Facilitators**  **ε : Barriers** | **α : Limitations**  **β : Perspectives** |
| Wagner, 2016 - Uganda | To compare the effects of two task-shifting models (clinical acumen vs protocolized care) of depression care on depression alleviation and antidepressant response | Cluster randomized study | Depression | 1252 patients | After training, nurses’ diagnoses, treat (antidepressant prescription) and follow-up patients with depression with validated questionnaires or clinical acumen during monthly visit.  At month 12 rates of depression alleviation were equivalent in the protocolized (75%) and clinical acumen (77%) | δ : training, supervision  ε : lack of mental health specialist | α : small number of clinics included, no control arm, costs not identified  β : nurses can provide quality depression care |
| Modula, 2018 - South Africa | To explore how nurses understood, interpreted, and implemented guidelines on mental health in HIV care center | Qualitative | Mental illness | Not detailed | Nurses viewed mental health screening and assessment as necessary processes to identify common mental health dis-  orders among HIV patients. They believed that both material and human resources were important in the assessment of mental health status. They verbalized different levels of  self-efficacy regarding holistic HIV management that included mental  health screening, with nurses without psychiatric training showing less  confidence. Their main concern was the  clients who do not show obvious signs of mental disorder. Therefore,  fundamental basic skills in assessment as well as interpersonal skills were viewed as essential in the detection of asymptomatic clients.  Therapeutic relations and good communication skills were identified as  important to encourage the clients to open and express their feelings | δ : number of nurses with  psychiatric nursing background needs to be increased, algorithms need  to be developed for mental health care (including short &  comprehensive tools for screening, assessment, and clear criteria for  referrals), development of continuous training  ε : lack of specifical tool to assess mental health | α : no details on study population  β : need for tool for effective management of mental illness by nurses |
| **First Author, Year - Location** | **Aim** | **Study Design** | **Disease** | **Sample** | **Results – Role or Knowledge of nurses** | **δ : Facilitators**  **ε : Barriers** | **α : Limitations**  **β : Perspectives** |
| Joubert, 2018 - South Africa | To explore the roles of psychiatric nurses at in-patient psychiatric facilities, the primary challenges associated with caring for mental health care users and the educational preparedness of nurses to deal with these challenges | Descriptive | Mental illness | 436 nurses | 48.4% of the respondents had completed a four-year Nursing diploma, about 30% a one-year Psychiatric Diploma. 92.2%; indicated that they preferred working at mental health care facilities.  Respondents identified substance abuse disorders (67.0%; n = 264), hallucinations (65.6%; n = 259) and delusions (60.2%; n = 238) as being the most frequent clinical and mental health problems that patients presented with.  The most common issues faced in psychiatric nursing practice were that “mental health care users deny mental illness” and the challenges associated with exposure to patients’ unpredictable behavior. Of significance was that nurses were also exposed to increased levels of aggression and violence. Psychiatric nurses reported  experiencing feelings of anger and frustration, as well as high levels of burnout. Psychiatric nurses indicated that they were suitably trained to deal with mental health care users, most supported the need for further training. | δ : continual education, training  ε : no identified | α : only include nurses working in psychiatric care  β : improve nursing curricula on mental health |
| Muga, 2019 - Kenya | To assess mental health literacy about depression with suicidal ideation among nurses in a private urban referral hospital in Kenya | Cross-sectional | Depression | 60 nurses | Nurses had low level of knowledge, 37 on 60 completed the questionnaire.  Three correctly identified the diagnosis of suicidal ideation and depression. 23 identified depressions only. Although 75% noted the symptoms were extremely distressing, fear, and stigma were common reactions. | δ : year of experience  ε : fear and stigma | α : small sample, only one private hospital  β : calls for improvement of knowledge and skills in diagnosis and treatment of depression with suicidal ideation |
| **First Author, Year - Location** | **Aim** | **Study Design** | **Disease** | **Sample** | **Results – Role or Knowledge of nurses** | **δ : Facilitators**  **ε : Barriers** | **α : Limitations**  **β : Perspectives** |
| Petersen, 2019 - South Africa | To evaluate a task shared integrated collaborative care package of care for chronic patients with co-existing depressive and alcohol use disorder symptoms | Repeated cross-sectional study | Depression | 1310 patients | Nurses after training worked as case manager, evaluated, and oriented patients according to the severity of symptoms to a trained nurse or psychiatrist.  There was a significant increase in the identification of depression and alcohol use disorder from pre-implementation to 12-month post-implementation. In the comparison group cohort study, patients with depressive symptoms having more than a 50% reduction in PHQ-9 scores were greater in the treatment group (n = 69, 55.2%) compared to the comparison group (n = 49, 23.4%) at 3 months (RR = 2.10, p < 0.001); and 12 months follow-up (intervention: n = 57, 47.9%; comparison: n = 60, 30.8%; RR = 1.52, p = 0.006). Remission (PHQ-9 ≤ 5) was greater in the intervention group (n = 32, 26.9%) than comparison group (n = 33, 16.9%) at 12 months (RR = 1.72, p = 0.016). | δ : training, supervision, use of a collaborative care package  ε : no identified | α : no random sampling, no control  β : a task shared collaborative stepped care model can improve detection and reduce depressive symptoms among patients with chronic conditions under real world conditions |
| Everitt-Penhale, 2019 - South Africa | To examine participants’ experiences of an adapted cognitive-behavioral therapy treatment for  adherence and depression, task-shifted and delivered by nurses  in two peri-urban HIV clinics near Cape Town | Qualitative | Mental Health | 2 nurses  9 patients | After training nurses performed behavioral therapy.  Participants indicated that the treatment as being effective in ameliorating their depressive symptoms and improving their adherence to treatment.  Additional benefits described included improvements in subjective wellbeing and social and occupational functioning. | δ : culturally adapted tool  ε : no identified | α : small sample, all data were not analyzed  β : culturally adapted tool will be interesting and acceptable to treat mental illness |
| Kemp, 2020 - South Africa | To identify patient-level factors that predicted successful detection of depressive symptoms by nurses, referral for depression-related treatment, and uptake of depression counseling, as part of integrated primary mental health care in  KwaZulu-Natal, South Africa | Retrospective records analysis | Mental Health | 412 patients | Nurses successfully detected depressive  symptoms in 208 [50.5%, 95% confidence interval (CI) 38.9–62.0] participants; of these,  they referred 76 (36.5%, 95% CI 20.3–56.5) for depression treatment; of these, 18 (23.7%, 95% CI 10.7–44.6) attended at least one session of depression counseling. Depressive symptom severity, alcohol use severity, and perceived stress were associated with detection. Similar factors did not drive referral or counseling uptake. Nurses detected patients with depressive symptoms at rates comparable to  primary care providers in high-resource settings, though gaps in referral and uptake persist. Nurses were more likely to detect symptoms among patients in more severe mental distress. | δ : depression severity, alcohol use, perceived stress  ε : limited referral possibilities | α : plausible misclassification of depression  β : implementation strategies for integrated mental health care in low-resource settings should  target improved rates of detection, referral, and uptake |
| Smith, 2020 - Rwanda | To assess implementation reach, fidelity, and clinical outcomes at health centers supported by The Mentoring and Enhanced Supervision at Health Centers for mental health during the scale up period | Before-and-after study | Mental health | 2239 patients | Mentoring and Enhanced Supervision at Health Centers was associated with high service use, improvements in mental health care delivery by primary care nurses, and significant improvements in clinical symptoms and functional disability of service users receiving care at health centers supported by the program | δ : training, supervision, use of algorithm, mentorship  ε : no identified | α : lack of control condition  β : Mentoring and Enhanced Supervision at Health Centers can reduce the evidence to practice gap for mental health care delivery by non-specialists in resource-limited settings |
| Kathree, 2023 – South Africa | To evaluate the viability of the real-world implementation of a collaborative care model on depression symptom reduction in chronic care patients based on nurse diagnosis and referral. | Comparison group cohort | Depression | 627 participants | Nurses were trained to identify and man- age depression in adult patients with chronic diseases using validated tools. Nurses independently assessed, diagnosed, and referred patients. Referral for treatment was independently associated with substantial improvements in depression symptoms three months later. | δ : availability of lay counselor trained to depression care  ε : lack of resources | α : observational design  β : nursing task-shared stepped-up collaborative care for depression treatment using co-located counselling in underserved real-world settings |
| **First Author, Year - Location** | **Aim** | **Study Design** | **Disease** | **Sample** | **Results – Role or Knowledge of nurses** | **δ : Facilitators**  **ε : Barriers** | **α : Limitations**  **β : Perspectives** |
| ***Chronic kidney diseases*** | | | | | | | |
| Gapira, 2020 - Rwanda | To assess the knowledge related to chronic kidney disease and perceived inpatient management among nurses at selected referral hospital in Rwanda | Descriptive | chronic kidney disease | 120 nurses | Eighty four percent (84%) had moderate level of knowledge related to chronic kidney disease and 51% of nurses had moderate level of perceptions regarding of inpatient management of chronic kidney disease. The factors associated with knowledge were institution type (p = 0.024), department (p = 0.000), level of education (p = 0.010) and type of specialty (p = 0.000). The factor associated perceived inpatient management of chronic kidney disease was department type (p = 0.015). A very weak non-significant positive relationship (r = 0.115, N = 120, p = 0.21) between knowledge and practice was observed. | δ : no identified  ε : no identified | α : low generalizability, plausible information bias  β : general nurses need to be given adequate evidence-based knowledge at university level to enable them to appropriately manage patients with chronic kidney disease |
| ***Chronic diseases globally*** | | | | | | | |
| Coleman, 1998 - South Africa | To describe the design and implementation of a nurse-led NCD service based on clinical protocols in a resource-poor area of South Africa | Retrospective records analysis | Diabetes, HBP, asthma, epilepsy | 1343 patients | Nurses diagnosed patients with new NCD, decided upon initial management in non-complex cases, continue to follow patient and adapt treatment and identified patients requiring referral to hospital.  Nurses controlled the clinical condition of 68% of patients with hypertension, 82% of those with diabetes, and 84% of those with asthma. Patient-reported adherence to treatment increased from 79% to 87% (p= 0.03) over the 2 years that the service was operating. | δ : nurse prescriptions were authorized, use of decision tree  ε : low resource of medications | α : study design  β : nurses effectively diagnose and manage non-complex cases of NCD, need to develop NCD’s primary care services managed by nurses |
| **First Author, Year - Location** | **Aim** | **Study Design** | **Disease** | **Sample** | **Results – Role or Knowledge of nurses** | **δ : Facilitators**  **ε : Barriers** | **α : Limitations**  **β : Perspectives** |
| Mamo, 2007 - Ethiopia | To describe the nurse led management of chronic disease in rural health center | Descriptive | Epilepsy, diabetes, cardiac disease and HBP | Not describe | Trained nursed, screen, treat and follow up treat for chronic diseases in rural health center. Referral is possible to the hospital. | δ : nurse training, partnership with a UK hospital, senior nurse coordinates the link between health center and hospital  ε : low resource of medications resulting in referral to hospital | α : study design  β : developing this model in other African countries |
| Kengne, 2009 - Cameroon | To contribute to strategies for the effective control and prevention of  commun NCDs | Prospective | Diabetes, HBP, asthma, epilepsy | 46 nurses | For each condition a training tool and a decisional algorithm was designed following international guidelines adapted to the country. The program was successful (high number of nurses trained, wide range of clinic tools developed, number of clinics set-up in each health district and the patients flow in pilot clinics) especially in rural area. | δ : effective nurse training, use of algorithm, low cost  ε : low provision in medications in rural area | α : no data provided to assess the performance of the clinics based on patients’ outcome  β : nurse-led clinics, algorithm driven service delivery stands as alternatives to overcome the shortage of trained physicians and other issues relating to access to care |
| **First Author, Year - Location** | **Aim** | **Study Design** | **Disease** | **Sample** | **Results – Role or Knowledge of nurses** | **δ : Facilitators**  **ε : Barriers** | **α : Limitations**  **β : Perspectives** |
| Katz, 2009 - South Africa | To explore questions related to the management of diabetes and HBP, by primary health care nurses in a dedicated chronic illness program for the primary care management of chronic diseases in Soweto and Southwest Gauteng region of Gauteng Province | Cohort | Diabetes, HBP | 2 nurses  618 patients | Nurses provided with decision support, escalated scaling up of medication, and prompt access to specialist care.  The program was successful in supporting nurses, detecting patients with advanced disease, and ensuring early referral to a specialist center. It improved early detection and referral of high risk, poorly controlled patients. | δ : complete program: training, decision tree, scaling up guidelines  ε : poor follow up due to poor existing health systems and the programs’ inability to integrate into existing chronic disease services | α : small sample, high rate of loss of follow up  β : clearly defined program improves nurses management of chronic disease |
| Parker, 2011 - South Africa | To evaluate the knowledge and practices of public-sector primary-care health professionals and final-year students regarding the role of nutrition, physical activity, and smoking cessation (lifestyle modification) in the management of chronic diseases of lifestyle within the public healthcare sector | Cross-sectional | Chronic diseases determinants | 149 nurses  61 doctors | Professional nurses had a mean score of 60 (SD 10) %, followed by nursing students (55 (SD 12) %), enrolled nurses (54 SD 12) %) and enrolled nursing assistants (49 (SD 12) %. In each of the test sections the doctors had the highest mean scores. Nursing staff had mediocre scores across most categories, with enrolled nursing assistants attaining the lowest mean scores, nurses and nursing students achieved poor scores compared with their perceived rating.  Barriers to prevention were: lack of time, lack of patient compliance and language barriers. | δ : no identified  ε : no identified | α : study conducted in the best-resourced province in South Africa  β : promote evolution of nurses’ curricula, improve continuing medical education |
| **First Author, Year - Location** | **Aim** | **Study Design** | **Disease** | **Sample** | **Results – Role or Knowledge of nurses** | **δ : Facilitators**  **ε : Barriers** | **α : Limitations**  **β : Perspectives** |
| Khabala, 2015 - Kenya | To assess the care of patients enrolled in medication adherence clubs’ | Cross-sectional | HBP  Diabetes  HIV | 1432 patients | Medication adherence clubs led by nurses enrolled 25-35 patients who meet quarterly to confirm clinical stability, talk with nurses, and received medication packs.  There were 1020 (71%) HIV and 412 (29%) non-communicable disease patients. Among those with NCD, 352 (85%) had hypertension and 60 (15%) had diabetes. During Medical adhesion clubs’ attendance, blood pressure, weight and laboratory testing were  completed correctly in 98–99% of consultations. Only 43 (2%) consultations required referral for clinical officer review. Loss to follow-up from the medical adhesion clubs’ was 3.5%. | δ : mutualization of chronic and infectious disease care  ε : no identified | α : selection bias linked to the lack of randomization  β : medication adherence clubs’ could help to support burden reduction and follow up of chronic disease patients |
| Maimela*,* 2015 - South Africa | To determine the perceptions and perspectives of chronic patients’ and nurses regarding chronic disease management in terms of barriers, facilitators, and their experiences | Qualitative | Chronic disease | 12 patients  10 nurses | Nurses disseminated health information in the community, organized semi-annual review of patients, weekly dedicated days for chronic conditions.  The main barriers are lack of knowledge on chronic diseases, shortage of medication and shortage of nurses in the clinics which causes patients to wait. Health care workers are poorly trained on the management of chronic diseases. Lack of supervision with poor dissemination of guidelines has been found to be a contributing factor to lack of knowledge in nurses. Mentioned the need to involve community health workers and traditional healers and integrate their services to early detect and manage chronic diseases in the community. | δ : organized management  ε : Limited availability of medication, equipment, and transport for nurses.  Shortage of nurses and other carers.  Lack of training for nurses. Lack of facilities for physical activity | α : purposive sampling  β : training and re-engineering of primary health care is a key point to improve access to chronic diseases care |
| **First Author, Year - Location** | **Aim** | **Study Design** | **Disease** | **Sample** | **Results – Role or Knowledge of nurses** | **δ : Facilitators**  **ε : Barriers** | **α : Limitations**  **β : Perspectives** |
| Malan, 2016 - South Africa | To evaluate the effect on clinical practice of training primary care providers in an approach to brief behavior change counselling | Before and after study | Chronic diseases globally | 23 nurses / nurse practionner  18 doctors | After training, nurses and doctors significantly changed their approach to counselling. Significant uptake of the counselling skills was seen immediately after training and 6-weeks later. | δ : training, cost-effective intervention  ε : no identified | α : did not measure clinical outcomes of patients  β : the training program should be integrated in nurses and doctors training |
| Some, 2016 - Kenya | To evaluate adherence to Médecins Sans Frontières clinical protocols when the care of five stable  NCDs (HBP, diabetes mellitus type 2, epilepsy, asthma, and sickle cell) was shifted  from clinical officers to nurses | Retrospective records analysis | HBP, diabetes, epilepsy, asthma, and sickle cell | 616 patients | After training, nurses done 733 consultations. Hypertension (64%, 397/616) was the most frequent NCD followed by asthma (17%, 106/616) and diabetes mellitus (15%, 95/616). Adherence to screening questions ranged from 65% to 86%, with an average of 69%. Weight and blood pressure measurements were completed in 89% and 96% of those required. Laboratory results were reviewed in 91% of indicated visits. Laboratory testing per NCD protocols  was higher in those with hypertension (88%) than diabetes mellitus (67%) upon review. 17 (2%) consultations were referred to clinical officers. | δ : guidelines, supervisions  ε : no identified | α : nurses could have falsely reported completing some of the  required tasks, and lack of referral back to a clinical officer does not exclude overlooked clinical, inability to assess long-term patient outcomes  β : Nurses can adhere to protocols for managing stable NCD patients based on clear  and standardized protocols and guidelines |
| **First Author, Year - Location** | **Aim** | **Study Design** | **Disease** | **Sample** | **Results – Role or Knowledge of nurses** | **δ : Facilitators**  **ε : Barriers** | **α : Limitations**  **β : Perspectives** |
| Ameh, 2017 - South Africa | To describe the viewpoints of operational managers and patients regarding quality of care in the integrated chronic disease management | Qualitative | Chronic diseases globally | 8 nurses  56 patients | For nurses: inadequacies in structure (malfunctioning blood pressure machines and staff shortage); process (irregular prepacking of drugs); and outcome (long  waiting times).Patients reported anti-hypertension drug stock-outs (structure); sub-optimal defaulter-tracing (process); rigid clinic appointment system (process). Emerging themes showed that patients reported HIV stigmatization in the community  due to defaulter-tracing activities of home-based carers. | δ : integrated model in already existent services  ε : stigmatization, shortage of nurses, waiting time, lack of material | α : purposive and small sample  β : Propose key for implementation in others low- and middle- income countries |
| Mahomed, 2017 - South Africa | To determine the perceptions and experiences of professional nurses with the integrated chronic care model that has been implemented in South Africa | Cross-sectional | Chronic diseases globally | 264 nurses | Prior to the implementation, 34% (91) of the staff perceived the model to be an added program whilst 36% (96) of the staff experienced an increased workload. Staff noted an improved process of care, better level of interaction with patients, improved level of knowledge and better teamwork coupled with an improved level of satisfaction with the work environment at the clinic after implementation of the integrated chronic disease model | δ : staff had positive perceptions, experiences  and attitudes towards the implementation of the Integrated Chronic Disease Management  ε : no identified | α : reasons for certain perceptions and experiences  were not explored, potential desirably bias  β : perceptions of staff are needed to effective implementation |
| **First Author, Year - Location** | **Aim** | **Study Design** | **Disease** | **Sample** | **Results – Role or Knowledge of nurses** | **δ : Facilitators**  **ε : Barriers** | **α : Limitations**  **β : Perspectives** |
| Sharp, 2020 - Eswatini | To assess the feasibility and impact of decentralized care for NCDs within nurse-led clinics in order improve access and inform healthcare planning in Eswatini and similar settings | Observational | HBP, diabetes | 1125 patients | There was a significant reduction in mean BP among HBP patients after four visits of 9.9 mmHg systolic and 4.7 mmHg diastolic (p = 0.01), and a non-significant reduction  in fasting blood glucose among diabetic patients of 1.2 mmol/l (p = 0.2). Key components of NCD care were  completed consistently by nurses throughout the intervention period, including a trend towards patients progressing from monotherapy to dual therapy in accordance with prescribing guidelines. | δ : training, guidelines  ε : loss of follow up, nurse rotation | α : observational data, high rate of loss of follow-up  β : the findings suggest that management of diabetes and hypertension care in a rural district setting  can be safely delivered by nurses |
| Niyonsenga, 2021 - Rwanda | To describe the fidelity, penetration, and feasibility of scaling-up nurse-led NCD clinic approach for severe NCDs | Retrospective review of cross-sectional | Diabetes, heart failure, severe HBP, severe chronic respiratory disease | 27,000 patients | After training, NCDs’ nurses diagnose, treat and follow-up patients for chronic diseases.  Hypertension was the most common diagnosis (70%), followed by type 2 diabetes (19%), chronic respiratory disease (5%), type 1 diabetes (4%) and heart failure (2%). Except for warfarin and beta-blockers, national essential medicines were available at more than 70% of facilities. Clinicians adhered to clinical protocols at approximately 70% agreement with evaluators. | δ : training, supervision by doctors, diagnostic and monitoring tool  ε : no identified | α : lack of baseline data of the availability of medications, equipment, and other resources  β : Nurse-led task-shifting has the potential to accelerate access to diagnosis and treatment for both severe and common NCDs |
|  |  |  |  |  |  |  |  |
|  |  |  |  |  |  |  |  |
| BP: Blood pressure ; HBP : High Blood Pressure ; HIV : human immunodeficiency virus ; HPV : Human papillomavirus ; HRQoL : Health – Related Quality of Life ; NA : not applicable ; NCD : noncommunicable disease ; SSA : Sub-Saharian Africa ; UK : United Kingdom ; VIA : visual inspection with acetic acid ; VILI : visual inspection with acetic acid and Lugol’s iodine | | | | | | | |
