# Supplementary material for: Role and knowledge of nurses in the management of non-communicable diseases in Africa: A scoping review
Source: PLoS One. 2024 Apr 18;19(4):e0297165. doi: 10.1371/journal.pone.0297165 (PMC11025970; doi:10.1371/journal.pone.0297165)
Supplement: Attachment — Submitted filename: Recommendation for the Reviewed Journal.docx [file pone.0297165.s005.docx]

**TITLE OF THE MANUSCRIPT**:

Role and knowledge of nurses in the management of non-communicable diseases in Africa: a scoping review

**RECOMMENDATIONS:**

1. **Grammar corrections:**

Line 85: Chronic diseases each year kills 31.4 million people in low .....

Line 96: and Embase between .. and April 2023

Line 145: To our knowledge ..

Line 148: To clearly?? sentence need to be re-written

Line 275: among them 3,009 were ....

Line 276: 455 articles that remained .....

Line 284: Eight (typo)

Line 295 - 298 I would rather use the word 'focusing on' .. instead of the word 'among'.

Line 303: among the 36 studies which focused on cancer, 12 were on breast cancer .......

Line 305: Studies on cervical cancer were focused on nurse-led ......

Line 316: for consistency, write 1 instead of one. (If you chose to use numerals instead of words then you should make sure you are consistent).

Line 323: exclusively represented ....

Line 325: misconceptions that ...

Line 329: were used.

Line 332: followed

Line 339: there was ..

Line 345: were focused

Line 352: What about the study which focused on nurse-led management of asthma? (this sentence is hanging, need clarification).

Line 385: To our knowledge, this was the first ....

Line 405: Our results showed ....

Line 441: Almost all of the included studies were conducted in English ....

**GENERAL RECOMMENDATION**

The authors clearly were careful in choosing this topic. The paper definitely provides a solution to a widely asked question on the role of nurses and other health care providers apart from physicians / medical doctors and medical specialists in the diagnosis and management of non-communicable diseases in Africa where they are fast increasing in prevalence due to various life changing landscapes.

However, the authors need to correctly write some of their sentences for them to have a clear meaning to the readers.

Also, the conclusion of the study which clearly stated what role can the nurses assume in these circumstances need to be supported by other strong findings which were not sated in this particular paper. Such issues as what training and for how long needs to be researched and explained in details for researchers to come to their stated conclusion.
