# Supplementary material for: Role and knowledge of nurses in the management of non-communicable diseases in Africa: A scoping review
Source: PLoS One. 2024 Apr 18;19(4):e0297165. doi: 10.1371/journal.pone.0297165 (PMC11025970; doi:10.1371/journal.pone.0297165)
Supplement: Attachment — Submitted filename: Response to Reviewers.docx [file pone.0297165.s006.docx]

Dear Delfina Fernandes Hlashwayo,

We would like to express our gratitude to you and the esteemed reviewers for dedicating valuable time and providing insightful feedback on our manuscript PONE-D-23-15682 entitled “Role and knowledge of nurses in the management of non-communicable diseases in Africa: a scoping review”.

Your constructive comments have undoubtedly enhanced the quality and rigor of our research, and we are honored by the opportunity to address these concerns and suggestions.

In this response letter, we have diligently addressed below each reviewer's comments, providing detailed explanations of the revisions we have made to the manuscript. The changes were highlighted in the manuscript.

**Editor comments :**

1. **Conduct thorough English proofreading to ensure that the manuscript adheres to proper grammar, style, and language usage.**

We performed a complete proofreading of the paper.

2. **Improve the clarity and alignment of the abstract with the objectives of the study.**

The abstract was modified in alignment with the objectives of the study: p3-4 L60-86.

3. **Enhance the introduction by addressing any inconsistencies and providing a clearer explanation of the role of nurses in the context of the study.**

We rewrite and completed the introduction : p4-6.

4. **Reconsider the inclusion of studies with low quality in your review. Evaluate their impact on the overall credibility and reliability of the findings. If included, we encourage you to provide a rationale for their inclusion or consider excluding them from the study.**

We added a paragraph in the Method section (L193-195) and the Limitations section (L359-369). It is important to note that the low-quality scores obtained by qualitative studies were due to the use of the Downs and Black scale, which was initially constructed for quantitative studies, this scale was employed as part of our concerted endeavor to standardize our evaluative approach. To enhance clarity, we removed the assessment of qualitive studies by the Downs and Black scale. We ensured the quality of qualitative studies using appropriate EQUATOR guidelines.

Furthermore, it is essential to clarify the nature of scoping reviews. Scoping reviews are a type of evidence synthesis that aims “to systematically identify and map the breadth of evidence available on a particular topic, field, concept, or issue, often irrespective of the source (i.e., primary research, reviews, non-empirical evidence) within or across specific contexts. Scoping reviews can clarify key concepts and definitions in the literature and identify key characteristics or factors related to a concept, including those related to methodological research”(Munn, 2022, JBI evidence synthesis). Moreover, regarding the assessment of the methodological quality of included studies, it is not a strict requirement for scoping reviews. However, we performed this assessment to elevate the quality of our research work and to provide a critical appraisal for readers regarding the level of evidence of the studies' findings. By conducting this assessment, we aimed to ensure transparency and rigor in our review process. Taken together, these arguments make it possible to maintain all the studies included in our review.

**In addition to these key points, please consider the following suggestions:**

1. **Please ensure to reference each supplementary file appropriately within the manuscript.**

It was done.

2. **To avoid redundancy, it would be advisable to keep the search terms in the supplementary file.**

It was done.

3. **Ensure that the information provided in Supplementary Table 2 is complete.**

The information provided in supplementary table 2 is complete.

4. **Please verify the accuracy and completeness of your reference list, including all relevant references, such as Higgins et al., 2019, and ensure consistent citation format throughout the manuscript as per the guidelines provided by the journal.**

We adjusted the reference list and verified citation format throughout the manuscript.

5. **We kindly request you to remove any unnecessary components of the main manuscript file, such as the Funding Statement, Declaration of Interests, Authorship Contribution, and Twitter handles. These can be included in the online submission system.**

It was done.

**Journal requirements:**

1. **Please ensure that your manuscript meets PLOS ONE's style requirements**

We modified the manuscript.

2. **In your Data Availability statement, you have not specified where the minimal data set underlying the results described in your manuscript can be found. PLOS defines a study's minimal data set as the underlying data used to reach the conclusions drawn in the manuscript and any additional data required to replicate the reported study findings in their entirety.**

S1 and S2 tables are available with the manuscript, they provide essential information that allows for the replication of the study findings.

3. **Please include a separate caption for each figure in your manuscript.**

It was done.

4. **We note that Figure 2 in your submission contain [map/satellite] images which may be copyrighted.**

We have modified Fig 2 using a non-copyrighted map from the public domain source: <https://commons.wikimedia.org/wiki/File:BlankMap-Africa.svg#filelinks>.

5. **Please include captions for your Supporting Information files at the end of your manuscript, and update any in-text citations to match accordingly.**

It was done: p26

**Reviewer #1:**

“**The authors clearly were careful in choosing this topic. The paper definitely provides a solution to a widely asked question on the role of nurses and other health care providers apart from physicians / medical doctors and medical specialists in the diagnosis and management of non-communicable diseases in Africa where they are fast increasing in prevalence due to various life changing landscapes.”**

Thank you for your positive comments.

**“However, the authors need to correctly write some of their sentences for them to have a clear meaning to the readers.”**

We performed a complete proofreading of the paper.

**“Also, the conclusion of the study which clearly stated what role can the nurses assume in these circumstances need to be supported by other strong findings which were not sated in this particular paper. Such issues as what training and for how long needs to be researched and explained in detail for researchers to come to their stated conclusion.”**

We modified the conclusion section (P16 L374-385) and precise in the results sections for training (P13 L297-298).

**Reviewer #2:**

**“I appreciate the opportunity to review the article Role and Knowledge of Nurses in the Management of non-communicable diseases in Africa: a scoping review.

This, without a doubt, is of interest for the development of nursing in the world, as well as it shows us a broad panorama of Nursing in Africa.

However, this document needs to be addressed in many aspects. I believe that if the researchers carry out an in-depth review of the document, as well as pay attention to all the comments, it can improve and be publishable in such a prestigious journal.

I comment on general points in case the researchers wish to attend to them and revise them.”**

Thank you for your positive comments. **“In relation to the summary: specifically the method, this is more than explaining what the researchers did, it must explain the design and search strategy, as well as the sources of information.**
**The conclusion of the summary does not correspond to the stated objectives, it does not describe the levels of knowledge they have and how they manage non-communicable diseases.
They show nurses as a solution to the lack of doctors, but not to the lack of nurses itself.”**

We modified the abstract according to these suggestions: P3-4 L60-86.

**“Background: page 6 line 125. Place an acronym, after the words: non-communicable disease (NCD).”**

It was done and the acronym was used throughout the manuscript.

**“Page 6 lines 139-143: it mentions twice that task-shifting is used, the paragraph is repeated and it does not mention what they are, only that it worked for HIV management.
At the same time, it does not justify that the lack of doctors is responded to by nurses taking on more activities if there is also a lack of personnel. They are not compensated and there is no mention of interdisciplinary work.”**

We modified this paragraph, we clearly describe the role of nurses for HIV and explained the role of nurses for chronic disease management as well as the fact that they work independently or collaboratively. (P5-6 L119-128)

**“Method: page 7, lines 158 to 163. It shows two types of appointments, it must be unified or in any case, for example. “According to Higgins(quote) in case you want to show the author's name.”**

It was modified.

**“Page 7 lines 168 -169. Much use is made of the first-person text, the passive voice is recommended in scientific articles.”**

We modified the method section using passive voice. (P6-9)

**“On the other hand, in this same section, it is observed that it is improper to show the degrees of the researchers or what they did when this is shown in the contributions of the researcher.”**

We withdrew the sentence.

**“Line 171-173. They could only mention the main topics of the review and mention the attached table in which it is specifically elaborated.
The search equations are removed from the method section and left as supplementary material.”**

It was done.

**“Page 10 method section.: The inclusion and exclusion criteria could be reduced to population and intervention, including in the last part of the objectives that were sought, such as level of knowledge.”**

We have modified this paragraph, but still using the PICOS method, which provides greater clarity from our point of view. (P7-8 L 161-175).

**“It does not include the years that are taken into account.”**

It was noted that “No language or publication date exclusion criteria were applied” (P8 L 173).

**“Page 11. Line 251 is peer review, not double blinding”**

Each investigator was unaware to the other's decisions regarding the inclusion of articles and throughout the other steps of the literature review, We modified the sentence. (P8 L179-180).

**“On pages 12 and 13 lines 295-299, I don't know if these results could make a table and point it out or if this comes in table 2, mention it.”**

Results are available in S2 Table, we mentioned it: P10 L227.

**“Page 13, line 303, You could mention the chronic diseases found or not communicable and later describe them.”**

We modified the paragraph but as the study focused on the different diseases we prefer immediately mentioned them. (P10 L231)

**“Page 15, Did the training last some time, was it given to all the nurses or specifically to a single group?** **Also, was the level of knowledge prior to the training assessed in any way? or how to prove that it worked?”**

It is difficult to summarize the content of all the training programs for all the studies in a few lines, so we have modified the paragraph and added some clarifications, as for the other studies, the study design and content of the training programs are available in the S2 Table. (P13 L 293 – 301)

**“Page 16, line 394: use passive voice**.”

It was done. (P14 L327)

**“Page 18 line 435 is missing an S.”**

It was modified.

**“Page 19 lines 453 to 459: Perhaps advanced practiced nurses because of the knowledge they have, but non-specialized nurses could not be a solution.”**

I agree with you but advanced practice nursing is not the only solution to address the healthcare challenges. Registered nurses, with appropriate training and support, can indeed play a crucial role in task-shifting strategies. By expanding the scope of practice for registered nurses and providing them with additional training in specific areas, they can effectively take on tasks traditionally performed by advanced practice nurses or physicians. Task-shifting to nurses is a viable and effective approach in various healthcare settings, especially in regions with limited healthcare resources, such as many parts of Africa.

We modified the conclusion. (P16 L374-386)

**“I recognize the supplementary material 2 summary table of the 111 articles is too extensive and has something that worries me a little. The document speaks in general about the knowledge that Nursing has, but in these articles, the population is doctors, nurses, and patients. Wouldn't it be exclusive to have different types of targeted populations? It seems that there was no systematization, but if this point is clarified, we will gladly continue.”**

We specified this point in the method (P7-8 L172-176). As described in the method, we included all studies that specifically examined nurses' involvement in chronic disease management or assessed nurses' knowledge related to chronic diseases. We excluded studies that did not provide explicit outcomes related to nurses to maintain a focused and relevant analysis. However, it is important to note that the studies we considered could involve not only nurses but also patients or doctors. As part of our comprehensive approach, we have reported the complete composition of the study population.

**“In supplemental material number 3. I consider that there are very low scores. Because those articles were not removed as they have poor methodological quality.”**

As explained above, very low scores were linked to qualitative design. We added a paragraph in the Method section (L193-195) and the Limitations section (L359-369). It is important to note that the low-quality scores obtained by qualitative studies were due to the use of the Downs and Black scale, which was initially constructed for quantitative studies. Despite receiving low scores, these qualitative studies were of good quality and adhered to the standards for qualitative research. This scale was employed as part of our concerted endeavor to standardize our evaluative approach. To enhance clarity, we removed the assessment of qualitive studies by the Downs and Black scale. We ensured the quality of qualitative studies using appropriate EQUATOR guidelines.

We believe that the revisions we have implemented significantly improve the overall clarity and impact of our work. The revised manuscript now aligns more closely with the high standards set by PLOS One, and we are confident that it will contribute meaningfully to the scientific community.

Once again, we express our gratitude for the guidance provided by the reviewers and yourself. We are committed to ensuring the highest quality of our research and welcome any further suggestions for improvement.

Please find attached the revised version of the manuscript.

Thank you for your continued support, and we eagerly await your decision on our revised submission.

Sincerely
