# Supplementary material for: Role and knowledge of nurses in the management of non-communicable diseases in Africa: A scoping review
Source: PLoS One. 2024 Apr 18;19(4):e0297165. doi: 10.1371/journal.pone.0297165 (PMC11025970; doi:10.1371/journal.pone.0297165)
Supplement: Attachment — Submitted filename: Reviwer comments Plos One.docx [file pone.0297165.s007.docx]

Reviwer’s comments

The authors have chosen a very interesting topic. The world is faced with a growing number of chronic diseases.

- In line 123, the sentence seems incomplete , I suggest that the authors complete the sentence
- In line 135 – well done to the authors -the review question is relevant in achieving the aim and objectives of the study.
- In terms of the results section, I suggest that the authors should start by describing the types of the studies reviewed, then present the reviewed results . the contents in line 210-219 may have been presented before the roles and knowledge of the nurses.
- It should have been of importance if the findings from the review were presented in themes , the reader of the manuscripts should know the types of roles and the knowledge found in the review.
- In line 282-300, the authors presented the barriers and facilitators ,where as in the aims of the study the authors talked about challenges and gaps. This creates a confusion. It feels like it is a two in one study. I suggest the authors be consistent with the words used.
- Line 315-324, the paragraph does not have reference, the starting of the paragraph starts with “To our knowledge …” which may show assumption , I believe there has been more reviews , this review may be adding to the available or bringing in new knowledge. I suggest that the authors look into paragraph.
- The study reviewed 111 studies, however in line 343-247,only two studies were included in relation to the barriers and facilitators.
