# Supplementary material for: Role and knowledge of nurses in the management of non-communicable diseases in Africa: A scoping review
Source: PLoS One. 2024 Apr 18;19(4):e0297165. doi: 10.1371/journal.pone.0297165 (PMC11025970; doi:10.1371/journal.pone.0297165)
Supplement: Attachment — Submitted filename: Response to Reviewers.docx [file pone.0297165.s008.docx]

Dear Delfina Fernandes Hlashwayo,

We would like to express our gratitude to you and the esteemed reviewers for dedicating valuable time and providing insightful feedback on our manuscript PONE-D-23-15682R1 entitled “Role and knowledge of nurses in the management of non-communicable diseases in Africa: a scoping review”.

Your constructive comments have undoubtedly enhanced the quality and rigor of our research, and we are honored by the opportunity to address these concerns and suggestions.

In this response letter, we have diligently addressed below each reviewer's comments, providing detailed explanations of the revisions we have made to the manuscript. The changes were highlighted in the manuscript.

**Editor comments :**

**General comments:**

1. **Please review the writing in the entire manuscript to ensure there are no grammatical errors, typos, or any other issues.**

It was done.

2. **Please review the abbreviation: NCDs stand for non-communicable diseases, while 'NCD' is used for the singular term.**

It was done.

**Methods:**

1. **Line 146: Consider capitalizing "PROSPERO" for consistency.**

It was done.

2. **Line 157: In the context of systematic reviews and database searches, the term "equation" is not commonly used. Instead, the more appropriate term is "search strategy."**

We have modified it.

3. **Lines 204-205: Please review the sentence construction for improved clarity.**

We modified this sentence: “Following the removal of duplicates, we screened a total of 3,464 articles. Of these, 3,009 were excluded as their titles and abstracts did not align with our specified criteria. Among the remaining 455 articles, a thorough review was conducted, leading to the identification of 111 articles that met our inclusion criteria and were subsequently included into this scoping review.”

**Results:**

1. **Line 225: Replace "6,3%" with "6.3%" for standard numeric representation.**

It was done.

2. **Line 304: Consider using "overall" instead of "globally" for better precision.**

We modified it.

3. **Reevaluate the placement of the quality assessment in the results section; it might be more suitable to position it elsewhere rather than at the end.**

We modified the order of the result section.

4. **Discussion: Please expand on the implications of your results for public health.**

We added a paragraph at the end of the discussion: “This study has important implications for public health. First, we emphasize the potential of task shifting to nurses to enhance healthcare access, particularly in regions with healthcare professional shortages. We also highlight the effectiveness of nurse-led chronic disease management, which supports the argument for efficient healthcare models, particularly in resource-constrained settings, where cost-effectiveness is paramount. Our research also indicates that nurse-led care can have a positive impact on patient satisfaction and outcomes, advocating for a shift towards patient-centered care models. This transformation is critical for improving management of chronic diseases. Nurse-led care also enhances continuity, ensuring that patients receive sustained support, education, and follow-up. This is of utmost importance in the effective management of chronic conditions and prevention of complications. Furthermore, our study highlighted the multifaceted role of nurses, extending beyond individual patient care to community health promotion. Nurses' involvement in addressing the risk factors for chronic diseases at the community level is crucial. Moreover, our research suggests potential avenues for the development of training programs and educational initiatives designed to equip nurses with the skills and knowledge necessary for effective chronic disease management. Furthermore, our study calls for policymakers in each country to formally define, recognize, and support nurses' roles in chronic disease management. This recognition could lead to the establishment of improved regulatory frameworks and increased resources for nurse-led care. Finally, our research contributes to data-driven decision-making within healthcare systems, enabling administrators to make informed choices regarding resource allocation.”

**Conclusions:**

1. **Consider beginning without the phrase 'despite these limitations'.**

It was done.

2. **Lines 383-384: Instead of "will guide," consider "serves as a guide for future research."**

We modified it.

3. **It is not customary to include citations in the conclusions section.**

We modified it.

**Figures and Supplementary files:**

1. **Fig 2: Please rephrase the caption for better clarity. Consider using a term other than "repartition" for improved adequacy.**

We modified this term by “distribution”.

2. **S2 Table: Consider repeating header rows for enhanced clarity.**

We modified it.

**Reviewer #2:**

**The authors have chosen a very interesting topic. The world is faced with a growing number of chronic diseases.**

Thank you for your positive comment.

**• In line 123, the sentence seems incomplete, I suggest that the authors complete the sentence**

We modified this sentence.

**• In line 135 – well done to the authors -the review question is relevant in achieving the aim and objectives of the study.**

Thank you for your positive comment.

**• In terms of the results section, I suggest that the authors should start by describing the types of the studies reviewed, then present the reviewed results. The contents in line 210-219 may have been presented before the roles and knowledge of the nurses.**

We modified the order in the results section.

**• It should have been of importance if the findings from the review were presented in themes, the reader of the manuscript should know the types of roles and the knowledge found in the review.**

Themes have been synthesized in the Discussion section, and we posit that pathology-specific synthesis is of paramount importance. This approach enhances the practicality and clarity of the results obtained from our research, particularly when considering the various pathologies addressed. This methodology enables the identification of interventions specific to each pathology in response to encountered issues, thus simplifying the process for the stakeholders. For instance, it is more straightforward to pinpoint mental health-related interventions in response to particular challenges than to seek general therapeutic education practices applicable across all contexts. Consequently, we have maintained the structure of the results section to reflect this comprehensive approach.

**• In line 282-300, the authors presented the barriers and facilitators, whereas in the aims of the study the authors talked about challenges and gaps. This creates a confusion. It feels like it is a two in one study. I suggest the authors be consistent with the words used.**

We modified the terms and standardize them.

**• Line 315-324, the paragraph does not have reference, the starting of the paragraph starts with “To our knowledge …” which may show assumption, I believe there has been more reviews, this review may be adding to the available or bringing in new knowledge. I suggest that the authors look into paragraph.**

We have revised the paragraph to present a more nuanced perspective, although we did not find any additional reviews on this specific topic. This section does not include references, as it serves as a synthesis of our research findings.

**• The study reviewed 111 studies, however in line 343-247, only two studies were included in relation to the barriers and facilitators.**

The two studies cited are not included in the review. These studies are used here to discuss the challenges and gaps identified in our review.

**Reviewer #3:**

**This manuscript provides a comprehensive overview to help guide the management of NCDs in Africa. There are a few recommendations.**

Thank you for your positive comment.

**In the introduction section, the authors should articulate the nursing roles for the management of NCDs as defined by nursing organizations within the country. This will enable a thorough discussion of this information with the study's findings.**

It is difficult to use country-specific definitions of nursing care. Indeed, there is not necessarily an authority regulating the nursing profession in every country, and because this is a global description, we wanted to use a universal international definition of nursing care.

**Additionally, it would be advantageous to provide a clear description of the levels of evidence present in the included studies.**

The quality scores of the included studies can be found in Table S3. It's important to note that, as a scoping review, the evaluation of study quality and level of evidence was undertaken to enhance the overall quality of our research and to offer readers a critical appraisal of the studies' findings. This assessment was carried out with the objective of ensuring transparency and rigor in our review process, although it is not considered a methodological mandatory requirement.

We believe that the revisions we have implemented significantly improve the overall clarity and impact of our work. The revised manuscript now aligns more closely with the high standards set by PLOS One, and we are confident that it will contribute meaningfully to the scientific community.

Once again, we express our gratitude for the guidance provided by the reviewers and yourself. We are committed to ensuring the highest quality of our research and welcome any further suggestions for improvement.

Please find attached the revised version of the manuscript.

Thank you for your continued support, and we eagerly await your decision on our revised submission.

Sincerely yours.
