# Supplementary material for: Role and knowledge of nurses in the management of non-communicable diseases in Africa: A scoping review
Source: PLoS One. 2024 Apr 18;19(4):e0297165. doi: 10.1371/journal.pone.0297165 (PMC11025970; doi:10.1371/journal.pone.0297165)
Supplement: Attachment — Submitted filename: Response to Reviewers.docx [file pone.0297165.s009.docx]

Dear Ermel Johnson,

We would like to express our gratitude to you and the esteemed reviewers for dedicating valuable time and providing insightful feedback on our manuscript PONE-D-23-15682R1 entitled “Role and knowledge of nurses in the management of non-communicable diseases in Africa: a scoping review”.

Your constructive comments have undoubtedly enhanced the quality and rigor of our research, and we are honored by the opportunity to address these concerns and suggestions.

In this response letter, we have diligently addressed below each reviewer's comments, providing detailed explanations of the revisions we have made to the manuscript. The changes were highlighted in the manuscript.

**Journal Requirements :**

1. **Please review your reference list to ensure that it is complete and correct. If you have cited papers that have been retracted, please include the rationale for doing so in the manuscript text, or remove these references and replace them with relevant current references. Any changes to the reference list should be mentioned in the rebuttal letter that accompanies your revised manuscript. If you need to cite a retracted article, indicate the article’s retracted status in the References list and also include a citation and full reference for the retraction notice.**

It was done. We added two references linked to the reviewers comments.

**Reviewer #3:**

1. **In the introduction section, the authors should articulate the nursing roles for the management of NCDs as defined by nursing organizations within the region. This will be consistent with the manuscript title and facilitates a comprehensive discussion of this information with the study's findings.**

We added a paragraph in the introduction, L130-145.

**Reviewer #4:**

1. **Thank you for addressing all the comments. The manuscript can be accepted for publication. The publication will assist other researchers on integrated management of communicable and non communicable diseases.**

Thank you very much.

We believe that the revisions we have implemented significantly improve the overall clarity and impact of our work. The revised manuscript now aligns more closely with the high standards set by PLOS One, and we are confident that it will contribute meaningfully to the scientific community.

Once again, we express our gratitude for the guidance provided by the reviewers and yourself. We are committed to ensuring the highest quality of our research and welcome any further suggestions for improvement.

Please find attached the revised version of the manuscript.

Thank you for your continued support, and we eagerly await your decision on our revised submission.

Sincerely yours.
